# Predicting the strength of urban‐rural clines in a Mendelian polymorphism along a latitudinal gradient

**DOI:** 10.1002/evl3.163

**Published:** 2020-03-25

**Authors:** James S. Santangelo, Ken A. Thompson, Beata Cohan, Jibran Syed, Rob W. Ness, Marc T. J. Johnson

**Affiliations:** ^1^ Department of Biology University of Toronto Mississauga Mississauga ON L5L 1C6 Canada; ^2^ Centre for Urban Environments University of Toronto Mississauga Mississauga ON L5L 1C6 Canada; ^3^ Department of Ecology and Evolutionary Biology University of Toronto Toronto ON M5S 3B2 Canada; ^4^ Department of Zoology and Biodiversity Research Centre University of British Columbia Vancouver BC V6T 1Z4 Canada

**Keywords:** Anthropocene, convergent evolution, parallel evolution, selection, urbanization

## Abstract

Cities are emerging as models for addressing the fundamental question of whether populations evolve in parallel to similar environments. Here, we examine the environmental factors that drive the evolution of parallel urban‐rural clines in a Mendelian trait—the cyanogenic antiherbivore defense of white clover (*Trifolium repens*). Previous work suggested urban‐rural gradients in frost and snow depth could drive the evolution of reduced hydrogen cyanide (HCN) frequencies in urban populations. Here, we sampled over 700 urban and rural clover populations across 16 cities along a latitudinal transect in eastern North America. In each population, we quantified changes in the frequency of genotypes that produce HCN, and in a subset of the cities we estimated the frequency of the alleles at the two genes (*CYP79D15* and *Li*) that epistatically interact to produce HCN. We then tested the hypothesis that cold climatic conditions are necessary for the evolution of cyanogenesis clines by comparing the strength of clines among cities located along a latitudinal gradient of winter temperature and frost exposure. Overall, half of the cities exhibited urban‐rural clines in the frequency of HCN, whereby urban populations evolved lower HCN frequencies. Clines did not evolve in cities with the lowest temperatures and greatest snowfall, supporting the hypothesis that snow buffers plants against winter frost and constrains the formation of clines. By contrast, the strongest clines occurred in the warmest cities where snow and frost are rare, suggesting that alternative selective agents are maintaining clines in warmer cities. Some clines were driven by evolution at only *CYP79D15*, consistent with stronger and more consistent selection on this locus than on *Li*. Together, our results demonstrate that urban environments often select for similar phenotypes, but different selective agents and targets underlie the evolutionary response in different cities.

**Impact Summary**

Investigating how populations have responded to similar environmental challenges helps us to predict future evolutionary responses in other taxa. Urban environments are a model for addressing the extent of parallel evolution in nature due to their convergent environments (e.g., heat islands, pollution, and fragmentation), such that two distant cities are often more similar to one another than either is to nearby nonurban habitats. In this paper, we used white clover (*Trifolium repens*) as a model to study the drivers of parallel evolution in response to urbanization. We collected over 11,000 plants from urban and rural habitats across 16 cities in eastern North America to examine how cities influence the evolution of a Mendelian polymorphism for an antiherbivore defense trait—hydrogen cyanide (HCN). This trait has previously been shown to exhibit adaptive evolution to winter temperature gradients at continental scales. Here, we tested the hypothesis that winter environmental conditions cause changes in the frequency of HCN on smaller spatial scales between urban and rural habitats. We found that half of all cities had lower frequency of HCN producing genotypes relative to rural habitats, demonstrating that cities drive parallel losses of HCN in eastern North America. We then used environmental data to understand what aspects of urban environments lead to reduced HCN frequencies. Warmer cities showed greater reductions in HCN frequencies in urban habitats, whereas colder, snowier cities showed little change in HCN between urban and rural habitats. This suggests that snow weakens the strength of natural selection against HCN in cities. However, it additionally suggests that alternative ecological or evolutionary mechanisms drive the strong differences in HCN between urban and rural habitats in the warmest cities. Overall, our work highlights urban environments as powerful, large‐scale models for disentangling the causes of parallel and nonparallel evolution in nature.

## Introduction

The extent to which populations adapt in parallel to similar environmental conditions remains a fundamental problem in evolutionary biology (Losos 2017; Bolnick et al. 2018). High levels of genetic and phenotypic parallelism suggest a limited number of adaptive evolutionary paths, increasing our confidence in predicting species’ responses to similar conditions (Losos 2011). Despite predictions that similar environments should select for similar alleles and phenotypes, the degree of parallelism observed both within and among species is often imperfect (Bolnick et al. 2018). Genetic constraints, genetic drift, and gene flow, among other processes, can all alter the amount of parallelism among populations (Bolnick et al. 2018; Langerhans 2018). To disentangle the many causes and consequences of parallel evolution in nature, we must leverage naturally repeated cases of adaption across habitat types (Steiner et al. 2009; Stuart et al. 2017; Langerhans 2018).

Urban environments are emerging as models for investigating the causes of (non)parallel evolution (*sensu* Bolnick et al. 2018) among natural populations (Rivkin et al. 2019). Cities tend to share many biotic and abiotic environmental variables such as increased temperatures, elevated pollution, greater habitat fragmentation, and altered structure and composition of ecological communities (McKinney 2006), which can drive parallel adaptive evolution (Reid et al. 2016; Winchell et al. 2016; Yakub and Tiffin 2017; Kern and Langerhans 2018). Additionally, the commonly observed decreased size and increased isolation of urban populations can drive parallel losses of genetic diversity within urban populations due to stronger genetic drift and restricted gene flow (Johnson and Munshi‐South 2017). Despite the many examples of parallel responses to urbanization, nonparallel responses—wherein phenotypes vary in the direction or extent of evolutionary change between replicate urban and nonurban populations—are also common (Thompson et al. 2016; Diamond et al. 2018). However, the causes of nonparallel responses to urbanization are currently poorly understood (Rivkin et al. 2019).

Recent work has used the globally distributed plant, white clover (*Trifolium repens*) as a model for examining (non)parallel evolutionary responses to urbanization. Thompson et al. (2016) documented repeated reductions in the frequency of hydrogen cyanide (HCN) within urban populations across three of the four cities examined in northeastern North America. HCN is known to deter feeding by herbivores (Santangelo et al. 2018b; Angseesing and Angseesing 1973; Saucy et al. 1999; Thompson and Johnson 2016; Dirzo and Harper 1982b), and gradients in the presence or intensity of herbivore pressure have been hypothesized to drive latitudinal and elevational clines in cyanogenesis in white clover's native and introduced ranges (Kooyers and Olsen 2013), due to the cost of producing HCN in the absence of herbivores (Kakes 1989). However, experimental work by Thompson et al. (2016) found no difference in herbivory along the urbanization gradient in Toronto, ON, prompting the authors to conclude that mechanisms other than reduced herbivore pressure in urban populations are driving the observed urban‐rural clines in cyanogenesis.

Observational and experimental data show that HCN is predicted to be costly in the presence of cold winter temperatures, either because such environments are associated with reduced herbivore pressure or because the metabolic components of HCN reduce tolerance to freezing (Daday 1954, 1965; Kooyers et al. 2018). Consistent with this latter prediction, some cities experience colder winter ground temperatures in urban populations that is correlated with reduced urban snow cover, leading to selection against HCN in cities (Thompson et al. 2016). The absence of a cline in one of the four previously studied cities was explained by high urban snow depth in both urban and rural locations, which was hypothesized to insulate plants against the damaging effects of frost (Thompson et al. 2016). If urban‐rural variation in snow depth is the only cause of urban‐rural clines in cyanogenesis, this leads to an explicitly testable prediction: cities lacking snow should lack cyanogenesis clines. The two previous studies that have documented such clines only sampled Canadian cities where minimum winter temperatures are below freezing (Thompson et al. 2016; Johnson et al. 2018), preventing a test of the hypothesis that colder winter conditions are the primary agent driving the evolution of urban clines in HCN. Sampling cities that vary in winter temperature and frost exposure is the logical next step to understand the environmental conditions under which we expect to find (non)parallel responses of HCN to urbanization.

Because neutral processes can sometimes drive parallel phenotypic responses in nature (Losos 2011; Bolnick et al. 2018), it is important to reject neutral explanations before inferring that parallel selection has led to repeated adaptive differentiation. Population genetic simulations suggests that although genetic drift could theoretically cause consistent clines if cities experience more drift (Santangelo et al. 2018a), empirical evidence from neutral microsatellite markers does not show evidence of more drift in urban clover populations (Johnson et al. 2018). Two additional lines of evidence would help to distinguish between the roles of selection and drift in generating phenotypic clines in HCN. First, although the two‐locus epistatic genetic architecture of HCN can lead to the evolution of clines due to drift (Santangelo et al. 2018a), neutral processes are expected to cause allele frequencies to vary randomly at the two underlying loci (Santangelo et al. 2018a). Thus, repeated clines in the same direction at individual loci underlying HCN can only be explained by selection driving urban‐rural population differentiation (Santangelo et al. 2018a). Second, an absence of clines in warm cities without snow would be consistent with altered selection in urban environments specifically caused by urban‐rural gradients in snow depth and minimum winter ground temperatures.

Identifying the target(s) of selection is crucial to understanding the traits that improve fitness in a given environment. Selection on linked genes outside the HCN pathway could give the misleading impression that selection is targeting HCN or either of its two component genes (*CYP79D15* and *Li*). Continental cyanogenesis clines in North America have arisen via sorting of pre‐existing and recurrent gene deletions at underlying loci (*CYP79D15* plus two downstream genes in the cyanogenic glucoside pathway, and *Li*) rather than novel mutations, such that multiple deletion haplotypes segregate at both loci in natural populations (Olsen et al. 2013; Kooyers and Olsen 2014; Olsen and Small 2018). Given the independent origin of these deletions, genes outside the HCN pathway are likely to be linked to only a single deletion haplotype at either underlying locus. Thus, the presence of multiple deletion haplotypes in both rural and urban populations would strengthen our inference that cyanogenesis is the target of selection rather than specific genes linked to the HCN loci; selection on genes linked to particular deletion haplotypes would be expected to lead to a single haplotype being under/overrepresented in urban or rural populations.

Here, we quantify urban‐rural clines in cyanogenesis in 16 large cities in eastern North America and leverage climatic variation among cities to test hypotheses about the causes of observed clines. We begin by assessing the environmental predictors of HCN frequencies along the latitudinal gradient writ‐large by asking: (1) what climatic variables predict mean HCN frequencies across latitude? Consistent with previous work (Daday 1954, 1965; Kooyers and Olsen 2012, 2013), we expected to observe lower HCN frequencies at more northern latitudes due to lower winter temperatures. We then focus on cyanogenesis clines in each city to address the following questions: (2) how common are urban‐rural cyanogenesis clines eastern North American cities? And (3) do regional environmental factors predict the strength of clines in cyanogenesis? We predicted that we would observe the weakest (or absence of) clines in cities with the warmest climates and also in those with the highest snowfall due to weaker frost‐mediated selection against cyanogenesis. Next, we investigate specific targets of selection by asking: (4) are clines present at both genes underlying cyanogenesis? Repeated clines in the same direction at either locus would indicate genetic drift does not cause urban‐rural clines in HCN. Clines at both loci or a single locus would be consistent with selection specifically acting on HCN or one of its component genes, respectively. Finally, we ask: (5) do urban populations show only a subset of the variation in deletion haplotypes as rural populations? The presence of equal numbers of deletion haplotypes segregating in urban and rural populations would suggest that selection is targeting HCN or its component genes, rather than linked sites. Our results highlight urban environments as large‐scale, replicated systems for addressing the ecological and genetic underpinnings of (non)parallel evolutionary responses in nature.

## Materials and Methods

### STUDY SYSTEM

White clover (*Trifolium repens* L., Fabaceae) is a perennial legume that reproduces clonally through the production of stolons and sexually through self‐incompatible, hermaphroditic flowers arranged in dense inflorescences (Burdon 1983). Plants are typically found in grazed or mowed pastures, lawns and meadows where they can maintain large and dense populations (Burdon 1983). Native to Eurasia, *T. repens* was introduced to temperate regions worldwide as a forage and nitrogen‐fixing crop (Burdon 1983; Kjærgaard 2003). Because of its long history of human‐mediated transport, white clover is found in cities all over the world, making it an ideal system for studying patterns of parallel evolution in response to urbanization.

Many white clover populations are polymorphic for the production of HCN, with cyanogenic (HCN present) and acyanogenic (HCN absent) cyanotypes co‐occurring (Daday 1958). The molecular genetics of the two individual loci underlying cyanogenesis has been recently characterized in detail (Olsen et al. 2008, 2007, 2013; Olsen and Small 2018). The *Ac/ac* polymorphism is caused by deletions overlapping the *CYP79D15* locus (hereafter *Ac*), which encodes the cytochrome P450 subunit involved in the synthesis of cyanogenic glycosides (linamarin and lotaustralin) stored in the cell vacuole (Olsen et al. 2008, 2013; Olsen and Small 2018). Plants require at least one functional allele with dominant expression (i.e., *Ac–*) to produce cyanogenic glycosides. Similarly, the *Li/li* polymorphism results from a deletion at the *Li* locus encoding the hydrolyzing enzyme linamarase, which is stored in the cell wall (Kakes 1985); the presence of at least one dominant allele (i.e., *Li*–) is required to produce linamarase. Thus, plants require a minimum of one dominant allele at each locus to produce HCN (i.e., cyanotype *Ac*– *Li*–), which is released when cell damage causes cyanogenic glycosides and linamarase to interact (Hughes 1991). If either locus is homozygous for the recessive allele, then a plant lacks HCN and is said to be “acyanogenic” (i.e., cyanotypes *Ac*– *lili*, *acac Li*–, and *acac lili*).

### SAMPLING AND HCN ASSAYS

**Figure 1:**
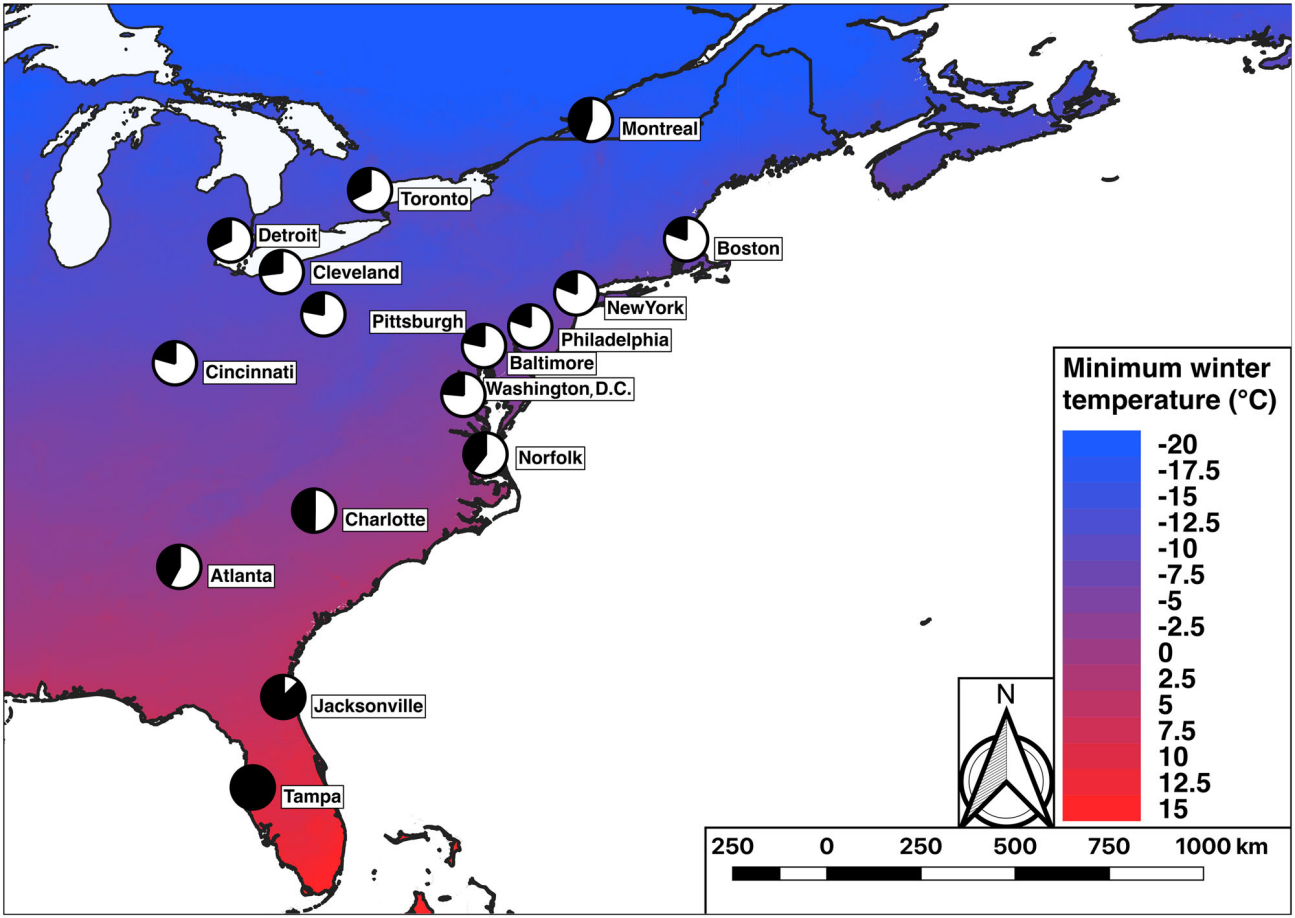
Map of 16 cities from which we sampled white clover populations along urban‐rural transects. Pie charts represent the mean frequency of HCN (black = HCN+; white = HCN−) for each city when averaged across all populations along the transect. Map color depicts the gradient in the minimum winter temperature (MWT, °C) taken from BioClim.

In May and June 2016, we sampled 15 plants from each of 15 to 45 populations (mean *n* = 38) along urban‐rural gradients in each of 12 cities in the eastern United States (Fig. 1). Sampling 15 plants resulted in a design that had similar statistical power as sampling 20 plants as in Thompson et al. (2016) (see Supporting Information text S2: “Power analyses for sampling design”). We sampled only large cities (240,000 < metropolitan human population size < 8,200,000; 151 < city area [km^2^] < 2300.) because these are likely to have the greatest environmental contrast between urban and rural habitats. We additionally chose cities along a north‐south latitudinal transect such that more southern cities had less snow and warmer winter ground temperatures, which previous research hypothesized might weaken selection against HCN in urban environments, leading to weaker or absent clines in southern cities (Thompson et al. 2016).

Sampling took place in three trips: trip one (May 16th to 23rd, 2016) involved collections from Tampa and Jacksonville, FL, Atlanta, GA, and Charlotte, NC; trip 2 (June 5th to 11th, 2016) from Norfolk, VA, Washington, D.C., and Baltimore, MD, and Philadelphia, PA; and trip 3 (June 15th to 20th) from Cleveland and Cincinnati, OH, Pittsburgh, PA, and Detroit, MI. In each city, we targeted populations spaced at least 1 km apart. In each population, we recorded the latitude and longitude coordinates and collected ∼6‐cm‐long white clover stolons with three to four intact leaves; stolons were at least 1.5 m apart to minimize sampling the same clonal genotype. Stolons were placed in sandwich bags and kept in a cooler with ice until being brought back to the lab where they were individually placed in 2 mL microcentrifuge tubes and stored at −80°C until HCN phenotyping. For plants collected during trip 3, we also visually estimated the amount of herbivore damage on two leaves per plant and used the mean of these measurements as our estimate of plant‐level herbivore damage (Johnson et al. 2016). In total, we collected and assayed 6738 stolons from 459 populations across 12 cities. For all downstream analyses, we combined the data from the 12 cities described above with the four cities (Boston, New York, Toronto, and Montreal) originally sampled by Thompson et al. (2016) using equivalent methodology. In total, we analyzed urban‐rural clines in HCN using 11,908 plants from 721 populations across 16 cities.

We used Feigl‐Anger assays to determine whether plants were cyanogenic or acyanogenic (Feigl and Anger 1966; Gleadow et al. 2011), which uses a simple color change reaction to determine the plant's phenotype. Briefly, we added a single mature leaf to wells in 96‐well plates with 80 μL of sterile deionized water. Leaf samples were added to alternating wells so that a single plate contained 48 plant samples. The plates were frozen at −80°C to facilitate cell‐lysis and release of HCN, and upon removal we macerated the plant tissue with pipette tips. We then covered the plate with Feigl‐Anger test paper and incubated the covered plate at 37°C for 3 h. Cyanogenic individuals (i.e., *Ac– Li–*) produce a blue spot on the filter paper above the well, whereas acyanogenic plants (i.e., *Ac– lili*, *acac Li–*, or *acac lili*) produce no color change.

To assess how clines in HCN are driven by changes in the frequency of the metabolic components of HCN (i.e., *Ac* or *Li*), we determined the presence/absence of *Ac* and *Li* for a subset of the cities (Atlanta, Baltimore, Charlotte, Cleveland, Jacksonville, New York, Norfolk, and Washington), which was combined with previously collected allele frequency information for *Ac* and *Li* for the city of Toronto (Thompson et al. 2016). For plants that tested negative for HCN, we added either (1) 30 μL of 10 mM exogenous cyanogenic glycosides (linamarin, Sigma‐Aldrich 68264) plus 50 uL of ddH_2_0 or (2) 80 μL of 0.2 EU/mL linamarase (LGC Standards CDX‐00012238‐100). A positive reaction in (1) indicates a plant produces linamarase (i.e., *acac Li*–); a positive reaction in (2) indicates a plant produces glycosides (i.e., *Ac*– *lili*); a negative reaction in both indicates plants that do not produce glycosides nor linamarase (i.e., the double‐homozygous recessive genotype, *acac lili*). These assays have been previously confirmed through PCR to reliably determine the cyanotype of individual *T. repens* genotypes (Olsen et al. 2008, 2007; Thompson and Johnson 2016). Due to the complete dominance of functional alleles at both loci, we cannot calculate the frequency of *Ac* or *Li* solely from the phenotyping assays described above (e.g., *AcAc* and *Acac* are indistinguishable). We therefore used the marker frequency of the homozygous recessive plants to calculate the frequency of *Ac* and *Li* assuming Hardy‐Weinberg Equilibrium (HWE, *p* ^2^ + 2*pq* + *q* ^2^ = 1), where *q* ^2^ represented the observed frequency of homozygous recessive genotypes at *Ac* (i.e., *acac*) or *Li* (i.e., *lili*). Although urban‐rural HCN clines may not always meet the assumptions of HWE (Santangelo et al. 2018a; Johnson et al. 2018), deviations from HWE are not predicted to greatly impact inferred allele frequencies when homozygous dominant and heterozygous individuals are phenotypically identical, as is the case for HCN (Lachance 2009; Kooyers and Olsen 2013). Analyzing changes in the frequency of *Ac* and *Li* for these nine cities allowed us to assess whether selection is targeting HCN specifically or individual loci underlying HCN production.

To examine whether urban and rural populations varied in the frequency of deletion haplotypes at *Ac* or *Li*, we used PCR with previously described forward‐reverse primer pairs (Olsen et al. 2014) to identify the relative size of deletions at both loci for individual plants. From the five or six most urban and rural populations, we extracted total genomic DNA from each of 10 randomly selected urban and rural plants (*n* = 20) for each of seven cities (Table 1) using a standard cetyltrimethyl ammonium bromide (CTAB)‐chloroform extraction method (Agrawal et al. 2012). We chose these cities because they spanned the range of latitudes included in our study, thus reducing potential impact of geographical variation on haplotype frequencies. We included cities that varied in the presence (five cities) and absence (two cities) of clines in HCN. We only extracted DNA from plants that were homozygous recessive at both loci (i.e., *acac lili*) because these plants have at least one deletion haplotype at each locus. Because no single primer pair spans the entire deletion, each plant was amplified with six different primer pairs (three for each locus), designed to assay the approximate size of the genomic deletion at each locus based on the presence/absence of PCR products (Kooyers and Olsen 2014). Note that larger deletions mask smaller deletions when resolving haplotypes on a gel, preventing us from estimating the true frequency of each deletion; we therefore used only the presence/absence of deletions in our analyses (see “Statistical analyses” below).

**Table 1:** Beta coefficients (i.e., slope) and *P‐*values from linear models testing the change in the frequency of HCN, *Ac*, or *Li* with increasing distance (standardized) from the urban center for each of 16 cities. Also shown are the total number of populations, the number of plants sampled in each city, and the mean frequency of HCN for the city. Bolded terms represent linear clines that were significant at *P* < 0.05. Boxes with dashes (–) represent cities where we did not quantify the frequency at the genes underlying HCN. Cities are arranged from north to south

City	Number of populations	Number of plants	Mean HCN frequency	*β*_HCN_	*β*_Ac_	*β*_Li_
Montreal	49	969	0.447	–0.057	–	–
Toronto↑	121	2379	0.323	**0.283** ***	**0.218** **	**0.271** ***
Boston	44	876	0.197	**0.119** *	–	–
Detroit	40	593	0.320	0.052	–	–
Cleveland	40	594	0.269	0.093	0.067	0.019
New York	48	946	0.191	**0.145** *	**0.204** *	0.033
Pittsburgh	40	590	0.221	0.069	–	–
Philadelphia	40	588	0.199	–0.031	–	–
Baltimore	39	584	0.216	0.031	0.065	0.031
Cincinnati	40	588	0.207	0.035	–	–
Washington, D.C.	45	658	0.236	**0.175** *	**0.326** **	–0.062‡
Norfolk	40	585	0.395	**0.337** ***	**0.358** ***	0.038
Charlotte	40	589	0.498	0.070‡	–0.077	–0.003‡
Atlanta	45	654	0.421	**0.362** ***	**0.263** **	**0.161** ‡, *
Jacksonville	35	500	0.872	**0.272** ‡, **	**0.202** **	**0.292** ‡, *
Tampa†	15	215	0.991	–0.029	–	–

Significance of *β* values:

^*^ *P* < 0.05; ^**^ *P* < 0.01; ^***^ *P* < 0.001.

^‡^Cities were better fit by a quadratic model (see online supplementary text S3: “Assessing the fit on nonlinear clines”) and showed a significant nonlinear change in the frequency of HCN, *Ac*, or *Li* with increasing distance from the urban center.

^†^Tampa was excluded from the analysis testing the environmental predictors of the slope of clines because it was effectively fixed for HCN (Fig. 1).

^↑^Number of populations and plants for Toronto reflects the total across three urban‐rural transects. The coefficients and *P*‐values here are from a model that includes all populations along the three transects because all three transects showed significant clinal variation when analyzed independently, and their slopes did not differ significantly from one another (Thompson et al. 2016).

Using the above approach, we were able to assign 88% and 78% of samples to previously described *Li* (*n* = 4) and *Ac* (*n* = 2) deletion haplotypes, respectively. The remaining individuals were either newly discovered haplotypes or individuals with intact *Li* and *Ac* genes, the latter indicating either false negative phenotyping, false positive haplotyping assays, or the presence of a silencer modifier locus. We focus our results on the haplotypes that aligned with those previously described by Kooyers and Olsen (2014) to understand whether one deletion haplotype versus multiple haplotypes were segregating in urban and rural populations in cities across our latitudinal transect.

### ENVIRONMENTAL DATA

To examine the regional abiotic factors that predict the strength of clines in HCN, we retrieved environmental data from open databases. First, we retrieved the minimum winter temperature (MWT, Bio6—an important predictor of HCN frequencies (Daday 1965; Kooyers and Olsen 2013) and the maximum summer temperature (MST, Bio5) using the highest resolution data (30 arc seconds; 1 km^2^) available from the BioClim database (version 1.4; Hijmans et al. 2005). We additionally retrieved the average monthly precipitation (Precip) at 1 km^2^ resolution from the same database. Next, we obtained the annual aridity index (AI), monthly average potential evapotranspiration (mPET), and average annual potential evapotranspiration (aPET) at 1 km^2^ resolution from the Consortium for Spatial Information (CGIAR‐CSI; Trabucco and Zomer 2009). These three abiotic factors are known predictors of the frequency of HCN and its component genes at continental scales in North America, Europe, and New Zealand (Kooyers and Olsen 2013). BioClim and CGIAR datasets are provided as gridded raster layers, from which we extracted the relevant data for all 721 populations using QGIS version v3.2.3 (QGIS Development Team 2018).

Finally, we obtained daily snow depth, snowfall, maximum temperature, and minimum temperature for all cities for the years 1980–2015 from the National Oceanic and Atmospheric Administration's National Centers for Environmental Information database (https://www.ncdc.noaa.gov/cdo-web/datatools/selectlocation). Importantly, these are regional environmental data obtained from a single weather station for each city located at the nearest international airport; thus, these data represent city‐level abiotic conditions and not the conditions extracted for each population.

Some filtering and processing of the environmental data was required prior to downstream analyses. First, we took the mean MWT (°C), MST (°C), AI, and aPET across all populations within a city to estimate the city‐level minimum winter temperature, maximum summer temperature, aridity, and annual potential evapotranspiration, respectively. Next, we calculated an alternative measure of aridity, the soil moisture deficit (SMD), as the difference between water supply (Precip) and water demand (mPET). Compared to the AI, which is a ratio and sensitive to fluctuate widely when numbers are small, SMD is more generally reliable (Thompson et al. 2014). We calculated SMD for all months from May to September to represent the plant growing season, and again took the mean of these measurements across all populations as our measure of city‐level SMD. Finally, we used NOAA's weather station data to estimate regional snow depth (cm), snowfall (cm), and the number of days where temperatures were freezing (<0°C) with no snow cover, a measure of frost exposure that has been previously linked to urban‐rural clines in HCN (Thompson et al. 2016). To retrieve these estimates, we first filtered the weather data to remove missing data and only included observations from January and February as these are the coldest winter months in eastern North America. We additionally removed observations from years where data were unavailable for both January and February and eliminated months with fewer than 10 days of weather data. Following filtering, we retained 31,005 weather observations (mean *n* = 1937 observations per city, Table S1). From these data, we took the mean snow depth, mean snowfall, and mean the number of days below 0°C with no snow cover across all years as our estimates of regional winter conditions.

### STATISTICAL ANALYSES

For brevity, we only briefly describe the statistical procedures used throughout the paper; a detailed description of all analyses can be found in the Supporting Information (text S1: “Detailed statistical analyses,” Table S2 and S3).

We first tested whether, on average, cities varied in mean HCN frequencies and whether urbanization influenced HCN frequencies. To do this, we fit an ANOVA using type‐III SS with within‐population HCN frequencies as the response variable and city, standardized distance to the urban center, and the city × distance interaction as predictors. We used distance to the urban center as a measure of urbanization as this is highly correlated with % impervious surface (*R* ^2^ = 0.64, Johnson et al. 2018) and sufficiently captures variation in HCN frequencies across urban‐rural gradients (Thompson et al. 2016; Johnson et al. 2018). Because urban‐rural transects varied in length, we scaled distance within cities between 0 (most urban) and 1 (most rural). In our model, a significant effect of city suggests that mean HCN frequencies vary across the 16 cities. A significant distance term means that across all cities, there is a significant urban‐rural cline in HCN frequencies, whereas a significant city × distance interaction indicates the strength or direction of clines in HCN varies across cities. For the four cities for which we collected herbivore damage data (Detroit, Cincinnati, Cleveland, and Pittsburgh), we fit models with the population‐mean herbivore damage as the response variable and standardized distance to the urban center as the sole predictor. None of these cities showed significant changes in herbivore damage across the urbanization gradient (see Supporting Information text S5: “Analysis of herbivore damage”) so we do not present these results any further.

To assess the environmental predictors of mean HCN frequencies across cities along our latitudinal gradient, we fit the following linear model: mean HCN frequency ∼ PC1_HCN_ + # days <0°C with no snow + AI + SMD, where PC1_HCN_ is a composite axis generated via principal components analysis (PCA) that explained 90.2% of the variation in MST, MWT, summer precipitation, annual PET, and snowfall, all of which were highly correlated and individually significantly predicted variation in HCN frequencies. Lower values of PC1_HCN_ represented cities with higher summer temperatures, higher minimum winter temperatures, higher summer precipitation, greater potential evapotranspiration, and lower snowfall. To obtain our final fitted model, we used a corrected Akaike Information Criterion (AIC_c_)‐based multi‐model selection and averaging process, whereby models with differing combinations of predictors were ranked by AIC_c_, and all models within 2 AIC_c_ units were averaged using the *dredge* function from the *MuMIn* package in R (Bartoń 2016).

Upon confirming that cities varied significantly in the strength of urban‐rural phenotypic clines using ANOVA, we used a similar approach to that described above to examine the environmental predictors of the strength of urban‐rural phenotypic clines in HCN. For each city, we first fit a linear regression with the proportion of cyanogenic plants within each population as the response variable and standardized distance to the urban center as the sole predictor. We extracted the slope (i.e., *β* coefficient) from each city's model as a measure of the strength of the clines and examined the environmental predictors of cline strength by running the following model: *β* ∼ PC1_slope_, where PC1_slope_ is a composite axis generated via PCA that explains 92.8% of the variation in snow depth, snowfall, MWT, and MST, all of which were highly correlated and on their own significantly predicted variation in the strength of clines (see text S1). Cities with low values along PC1_slope_ experience little snow, higher minimum winter temperature, and higher maximum summer temperature. For consistency with previous literature examining the maintenance of gene frequency clines by natural selection (Endler 1977), we replicated this analysis by fitting logistic regressions to the individual plant phenotype data and examining the environmental predictors of the change in log‐odds extracted from such models. The results mirrored those from the population‐mean linear regressions so we report the latter in the main text to be consistent with recent literature examining urban‐rural clines in cyanogenesis (Thompson et al. 2016; Johnson et al. 2018). Results from the logistic regressions are reported in the Supporting Information (text S4: “Logistic regressions on individual plant data,” Table S4 and Fig. S1–S6).

Finally, we explored whether clines were present at each of the two loci underlying HCN. We fit linear models in which the allele frequencies for the *Ac* and *Li* loci were treated individually as response variables in separate analyses, with standardized distance to the urban center as the sole predictor. To examine variation in deletion haplotypes across urban and rural habitats, we used the raw counts of deletion haplotypes at each locus to calculate the haplotype richness (i.e., number of unique haplotype deletions) in urban and rural habitats for each city. We fit haplotype richness as the response variable in an ANOVA with habitat type (i.e., urban vs. rural) as the sole predictor such that a significant effect of habitat suggested differences in deletion haplotype richness among urban and rural habitats. All analyses were performed in R version 3.6.1 (R Core Team 2019).

## Results

### VARIATION IN HCN FREQUENCIES IN CITIES ALONG A LATITUDINAL GRADIENT

**Figure 2:**
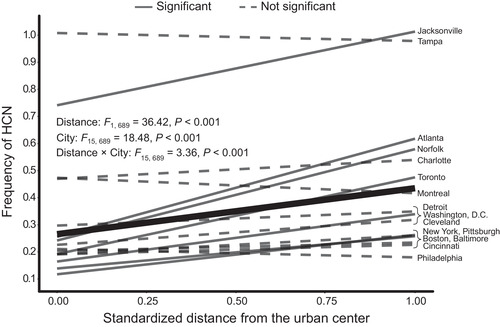
Urban‐rural clines in the frequency of HCN within populations of *Trifolium repens* across 16 cities in eastern North America. The frequency of HCN within *T. repens* population is plotted against the standardized distance from the urban center. Solid lines represent linear regressions from cities where the phenotypic cline in HCN was significant at *P* < 0.05, whereas dashed lines are cities that lack significant clinal variation. The thick black line represents the main effect of standardized distance on HCN frequencies, averaged across all cities.

**Figure 3:**
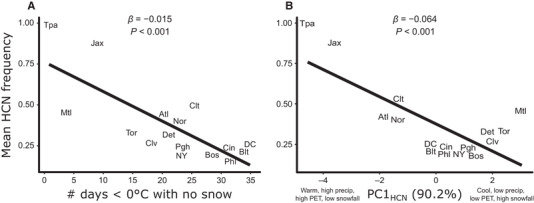
Mean HCN frequency across cities was influenced by (A) the number of days below 0°C with no snow cover—a measure of exposure to frost—and (B) PC1_HCN_, a component axis accounting for 90% of the variation in maximum summer temperature (°C, Bio5), minimum winter temperature (°C, Bio6), annual potential evapotranspiration (mm), monthly summer precipitation (mm), and snowfall (cm). City labels are slightly jittered to avoid overlap, if necessary. Cities with low values along PC1_HCN_ have relatively high summer temperatures, high minimum winter temperatures, high summer precipitation and potential evapotranspiration, and low snowfall, whereas cities with high values along PC1_HCN_ have the opposite. (City abbreviations: Jacksonville [Jax]; Tampa [Tpa]; Atlanta [Atl]; Norfolk [Nor]; Charlotte [Clt]; Toronto [Tor]; Montreal [Mtl]; Detroit [Det]; Washington, D.C. [DC]; Cleveland [Clv]; New York [NY]; Pittsburgh [Pgh]; Boston [Bos]; Baltimore [Blt]; Cincinnati [Cin]; Philadelphia [Phl]).

The mean frequency of HCN varied across the 16 cities from 19% (New York) to 99% (Tampa) (main effect of city: *F*_15,689_ = 18.48, *P* < 0.001, Table 1, Figs. 1 and 2), with the highest frequencies at the most southern latitudes (Fig. S7). The number of days <0°C with no snow cover and PC1_HCN_ together accounted for 94.1% of the variation in mean HCN frequencies among cities (Table S5). Specifically, HCN frequencies decreased by 1.5% for every additional day below 0°C with no snow cover (*β* = −0.015 ± 0.002 SE, *z* = 8.77, *P* < 0.001, Table S6, Fig. 3A), and by 6.4% for every unit increase along PC1_HCN_ (*β* = −0.064 ± 0.008 SE, *z* = 7.0, *P* < 0.001, Table S6, Fig. 3B). Neither annual AI nor soil moisture deficit was strong predictor of mean HCN frequencies in our model (Table S6).

### ENVIRONMENTAL PREDICTORS OF URBAN‐RURAL CLINES IN HCN FREQUENCIES

**Figure 4:**
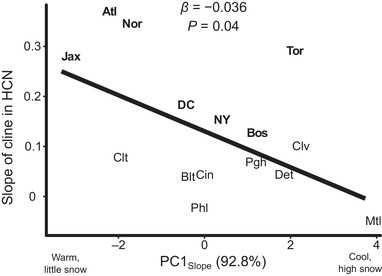
The strength of urban‐rural clines in HCN was influenced by PC1_Slope_, a composite axis that accounts for 93% of the variation in minimum winter temperature (°C, bio6), maximum summer temperature (°C, bio5), snowfall (cm), and snow depth (cm). City labels are slightly jittered to avoid overlap. Bolded cities shower significant linear changes in HCN along urbanization gradients. Cities with low values along PC1_Slope_ have relatively little snow and higher minimum winter and maximum summer temperatures, whereas cities with high values along PC1_Slope_ have the opposite. Abbreviations are the same as in Figure 3.

On average, urbanization was associated with reduced HCN frequencies across cities, whereby the main effect of standardized distance from the urban center was positively associated with the frequency of HCN within *T. repens* populations (main effect of distance, *F*_1,689_ = 36.42, *P* < 0.001, Fig. 2). In a model with unstandardized distance as a predictor, this translated into an average increase in HCN frequency of 0.3 % per km from the urban center. Critically, the slope of urban‐rural clines in HCN varied across cities (distance × city interaction: *F*_15,689_ = 3.26, *P <* 0.001, Table 1, Fig. 2). The strength of urban‐rural clines decreased with increasing values along PC1_slope_ (*β* = −0.036 ± 0.016 SE, *t*_13_ = −2.27, *P* = 0.04, *R* ^2^ = 28%, Fig. 4), implying that the strongest clines occurred in cities with the warmest environments and the weakest clines occurred in cities with low temperature and high snowfall/snow depth.

### CLINES AT LOCI UNDERLYING HCN AND DELETION HAPLOTYPE FREQUENCIES

We assayed the genotype at the two loci underlying cyanogenesis in nine (of 16) cities, six of which showed significant clines in HCN (Table 1). Of the six cities with significant clines, three (Atlanta, Jacksonville, and Toronto) had significant linear clines at both *Ac* and *Li*, three (New York, Norfolk, and Washington) had significant linear clines only at *Ac*, and no cities had a significant linear cline only at *Li*. Significant clines at *Ac* and *Li* were always in the same direction as clines in HCN (i.e., lower frequencies of the dominant alleles *Ac* and *Li* in urban populations). None of the three cities that lacked a cline in HCN showed a cline at either underlying gene.

All deletion haplotypes at *Ac* and *Li* identified previously in this system were found in each city. Haplotype richness (*Ac*: Richness_rural_ = 2 ± 0 SE, Richness_urban_ = 1.86 ± 0.14 SE, *t*_11_ = 0.92, *P =* 0.37; *Li*: Richness_rural_ = 3.67 ± 0.21 SE, Richness_urban_ = 3.14 ± 0.34 SE, *t*_11_ = 1.26, *P =* 0.24) did not vary between urban and rural habitats at either locus. These results suggest that no specific deletion haplotype is favored in urban habitats at either *Ac* or *Li*.

## Discussion

We combined field sampling of white clover populations from large eastern North American cities with environmental data to assess the environmental correlates of cyanogenesis on a continental scale and of urban‐rural gradients in HCN frequencies. Several key results are most important to answering our research questions. As expected, HCN frequencies increased southward across the continent as frost exposure decreased (question 1). Among cities that were not effectively fixed for HCN, urban‐rural cyanogenesis clines have evolved in approximately half of the cities studied (question 2) and the steepest clines occurred in the warmest environments (question 3). Clines in HCN were matched by clines at one or both of the loci underlying HCN, and these clines were always in the same direction (question 4). Finally, the richness of deletion haplotypes among acyanogenic plants was consistent across urban and rural populations of multiple cities (question 5). Together, these results provide compelling evidence that selection is driving the parallel evolution of cyanogenesis clines across multiple large urban centers in North America, although regionally cold and snowy climates dampen parallel responses of HCN to urbanization. Below, we discuss our results in the context of the environmental drivers of evolution and the parallel evolution of urban‐rural clines.

### ENVIRONMENTAL PREDICTORS OF HCN FREQUENCIES

The cyanogenesis polymorphism has long served as a model for assessing the climatic drivers of adaptation in natural populations. Our results are consistent with previous work demonstrating a cost of producing HCN or its metabolic components in frost‐prone habitats (Daday 1954, 1958, 1965; Ganders 1990; Kooyers and Olsen 2013; Kooyers et al. 2018); northern cities with lower winter temperatures and greater frost exposure had lower cyanogenesis frequencies than southern cities. In contrast to previous work (Kooyers and Olsen 2013), we did not identify aridity as an important predictor of mean HCN frequencies, possibly because the latitudinal transect sampled here spanned a shallow aridity gradient (annual AI range: 0.84–1.22) and steeper gradients may be necessary to detect aridity as an important correlate of HCN frequencies (e.g., New Zealand cline, AI range: 0.5941–4.8569, Kooyers and Olsen 2013).

### SELECTION VERSUS DRIFT IN THE EVOLUTION OF URBAN‐RURAL CYANOGENESIS CLINES

Two lines of evidence presented here solidify the role of selection rather than drift in driving the evolution urban‐rural HCN clines. First, the presence of repeated clines in the same direction at individual loci underlying HCN strongly implicates selection because genetic drift is expected to drive random fluctuations in allele frequencies along spatial clines at a single locus (Santangelo et al. 2018a). Second, the negative relationship between the strength of clines and regional winter conditions suggests that latitudinal variation in winter temperature and snow depth—or something correlated with it—modulates the strength of selection along urban‐rural gradients, driving phenotypic clines in cyanogenesis. Additionally, the equivalent deletion haplotype richness in urban and rural populations across multiple cities suggests that selection is targeting HCN or its component genes rather than genes tightly linked to particular deletion haplotypes.

### PARALLEL EVOLUTION OF URBAN‐RURAL CLINES IN CYANOGENESIS

We detected clines in HCN in roughly half (47%; 7/15) of sampled cities, suggesting levels of phenotypic parallelism in this system are at best imperfect. Indeed, (non)parallel evolution appears common across systems and may occur for a number of reasons including genetic drift, gene flow, and variation in the presence or strength of putative selective agents, among others (Bolnick et al. 2018; Langerhans 2018). Due to the stochastic loss of alleles in small populations, genetic drift can reduce parallelism by lowering the likelihood that replicate populations contain the same beneficial alleles, and reducing the efficacy of selection to fix those alleles (MacPherson and Nuismer 2017). However, the epistatic genetic architecture of HCN makes populations particularly prone to loss of HCN via drift (Santangelo et al. 2018a), rather than causing changes in random directions (Colautti and Lau 2015). This deterministic outcome of drift, when coupled with the frequently observed reduction in effective population size in cities (Johnson and Munshi‐South 2017), suggests that genetic drift may *increase* rather than decrease levels of parallelism in this system. Although investigation of how urbanization influences the strength of genetic drift is certainly warranted, variation in the strength of drift across cities is unlikely to explain the imperfect parallelism observed in the present study.

Gene flow between divergent habitats can constrain local adaptation, reducing levels of parallelism when the magnitude of this effect varies across replicate populations (Stuart et al. 2017; Bolnick et al. 2018; Langerhans 2018). Recent population genetic analyses suggest urban‐rural clines in cyanogenesis are evolving despite substantial gene flow between urban and nonurban populations (Johnson et al. 2018). In addition, the smooth sigmoidal clines observed in the present study (Figs. S2 to S5) suggest that gene flow may be constraining the formation of urban‐rural clines in HCN, because strong gene flow tends to increase the width of clines and lead to more gradual changes in gene or allele frequencies across space (May et al. 1975; Endler 1977). If cities vary in the extent of gene flow between urban and nonurban populations, then the formation of clines may be more constrained in some cities than others, leading to reduced parallelism. Rates of gene flow between urban and nonurban white clover populations are unknown, and the constraining effect of gene flow will additionally depend on the differential fitness of cyanogenic and acyanogenic genotypes at opposing ends of the urbanization gradients. There is therefore ample room for both genomic and experimental work to assess the relative contributions of gene flow and selection to the formation and maintenance of urban‐rural cyanogenesis clines.

Environmental heterogeneity among “replicate” environments can lead to variation in the strength of selection favoring particular phenotypes, potentially reducing the extent of parallel evolution (Stuart et al. 2017). Previous work in white clover identified exposure to colder winter temperatures in urban populations as a putative mechanism driving reduced HCN frequencies in urban environments, which was alleviated in cities with high snowfall (Thompson et al. 2016). Based on this earlier work, we predicted the weakest clines in cities with high mean winter temperature or high snowfall due to the absence of frost‐mediated selection against HCN in these cities. Consistent with this prediction, cities with high snowfall (i.e., high values along PC1_slope_) had the weakest clines, potentially due to snow buffering plants from frigid temperatures and weakening frost‐mediated selection against HCN‐producing genotypes. However, the strongest clines occurred in the warmest cities (i.e., low values along PC1_slope_), which refutes our hypothesis that regional snow cover is necessary for urban‐rural clines in cyanogenesis to evolve. Importantly, this provides no information about whether lower winter temperatures in cities is an important selective agent; urban frost may still be important in frost‐prone cities with shallower urban‐rural gradients in snow depth, as suggested by currently available data (Thompson et al. 2016). However, this does suggest that alternative mechanisms must drive the evolution of clines in warmer cities where frost is uncommon. Indeed Atlanta, which gets little snow (mean snowfall = 0.07 cm/year) and is relatively warm throughout the winter months (mean minimum winter temperature = −0.43°C), had the strongest of all observed urban‐rural cyanogenesis clines (*β* = 0.36, Table 1). Together, these results demonstrate that although urban and rural populations may appear superficially similar, regional (and likely local) factors are contributing to variation in putative selective agents across habitat types and cities, thereby reducing levels of parallelism.

The occurrence of steeper clines in warmer cities suggests that regional temperature modulates the strength of selection along urban‐rural clines in some cities. Given that cyanogenesis functions as an antiherbivore defense (Santangelo et al. 2018b; Thompson and Johnson 2016; Dirzo and Harper 1982a), some clines could be explained by differential herbivory among urban and rural populations if urbanization reduces herbivore populations (Raupp et al. 2010; Miles et al. 2019; Moreira et al. 2019). Although previous experimental work found a negligible role of herbivory as a driver of urban clines in HCN (Thompson et al. 2016), this work was performed in a single northern city (Toronto). Because herbivory often increases with warmer temperatures (Lemoine et al. 2014), the role of herbivory in generating urban‐rural clines in HCN may be more important in warmer, southern cities. Additional work quantifying the strength of clover‐herbivore interactions, and biotic interactions more generally, in these cities is needed.

Incidences of parallel phenotypic evolution may not always be mirrored by parallel genetic responses. Indeed, we expect levels of genetic parallelism to decrease with an increasing number of genes contributing to a phenotype because there are an increasing number of ways of achieving the same phenotypic outcome (Conte et al. 2012; MacPherson and Nuismer 2017). This may be especially true if underlying genes have alternative functions or contribute pleiotropically to other phenotypes that may be under selection. In the two‐locus HCN system, we found that some phenotypic clines were mirrored by clines at both underlying genes, suggesting selection is acting on HCN. By contrast, other clines were mirrored only by clines at *Ac*, suggesting selection may be targeting only the production of cyanogenic glucosides. Recent experimental data suggest a cost to producing the metabolic components of HCN in stressful environments, especially for cyanogenic glucosides (Kooyers et al. 2018). Therefore in some cases, selection may be acting on this locus due to its greater costs in stress‐prone environments (Kooyers et al. 2018), independent of its involvement in HCN production.

## Conclusions and Future Directions

We have demonstrated the repeated evolution of urban‐rural cyanogenesis clines across eastern North American cities. A major goal for future work in this system entails distinguishing among the targets of selection across replicate clines (i.e., *HCN* vs. *Ac*. vs. *Li*) and disentangling the numerous ecological (e.g., environmental factors) and evolutionary (e.g., selection and drift) drivers of (non)parallel responses of HCN to urbanization. This work will require quantifying a broad array of environmental factors at a finer scale (e.g., population level) in cities spanning all continents, and would benefit from exploring whether clines exist at other loci across the genome. White clover is a natural model for understanding how cities drive parallel evolution on a global scale due to its ubiquity across the globe and ease of sampling and phenotyping. Such work would advance our understanding of how cities influence the evolution of populations in our own backyards, and further cement the utility of cities as useful models for understanding the causes and consequences of parallel evolution in nature.

## CONFLICT OF INTEREST

The authors declare no conflict of interest.

Associate Editor: Z. Gompert

## Supporting information


**Table S1**. Minimum and maximum years, and total number of weather observations for the months of January and February across all 16 cities.
**Table S2**. Pearson product‐moment correlation coefficients (lower triangle) and associated *P*‐values (upper triangle) for all pairwise combinations of environmental variables collected for our analyses.
**Table S3**. Best‐fit model for the change in within‐population HCN, *Ac*, or *Li* frequencies along our urbanization gradient.
**Table S4**. Beta‐coefficients, standard errors, z‐statistics, and *P*‐values from binomial logistic regressions for each city using the individual plant phenotype data (i.e., HCN+ = 1; HCN− = 0) as the response variable, and standardized distance to the urban center as the sole predictor.
**Table S5**. Top two models with ΔAIC_c_ < 2 returned from the model selection performed using ‘dredge’ in R.
**Table S6**. “Full” model averaged coefficients from the model selection and averaging of top models with environmental predictors of mean HCN frequencies.
**Figure S1**. Urban‐rural clines in the frequency of HCN within populations of *Trifolium repens* across 16 cities in eastern North America.
**Figure S2**. Logistic clines fit to (a) Atlanta, (b) Baltimore, (c) Boston, and (d) Charlotte using the individual plant level phenotype data (i.e., HCN+ = 1; HCN− = 0) as the response variable (y‐axis) and standardized distance to the urban center as the sole predictor (x‐axis).
**Figure S3**. Logistic clines fit to (a) Cincinnati, (b) Cleveland, (c) Detroit, and (d) Jacksonville using the individual plant level phenotype data (i.e., HCN+ = 1; HCN− = 0) as the response variable (y‐axis) and standardized distance to the urban center as the sole predictor (x‐axis).
**Figure S4**. Logistic clines fit to (a) Montreal, (b) New York, (c) Norfolk, and (d) Philadelphia using the individual plant level phenotype data (i.e., HCN+ = 1; HCN− = 0) as the response variable (y‐axis) and standardized distance to the urban center as the sole predictor (x‐axis).
**Figure S5**. Logistic clines fit to (a) Pittsburgh, (b) Toronto, and (c) Washington, D.C. using the individual plant level phenotype data (i.e., HCN+ = 1; HCN− = 0) as the response variable (y‐axis) and standardized distance to the urban center as the sole predictor (x‐axis).
**Figure S6**. The strength of urban‐rural clines in HCN—measured here as the change in the log‐odds that a plant is cyanogenic with increasing distance to the urban center (y‐axis)—was influenced by PC1_SlopeLog_, a composite axis that accounts for 87% of the variation in annual PET, monthly precipitation, minimum winter temperature, maximum summer temperature, snow depth, and snowfall.
**Figure S7**. Mean frequency of HCN within a city as a function of latitude.
**Figure S8**. Map of the urban‐rural transect for the city of Atlanta.
**Figure S9**. Map of the urban‐rural transect for the city of Baltimore.
**Figure S10**. Map of the urban‐rural transect for the city of Boston.
**Figure S11**. Map of the urban‐rural transect for the city of Charlotte.
**Figure S12**. Map of the urban‐rural transect for the city of Cincinnati.
**Figure S13**. Map of the urban‐rural transect for the city of Cleveland.
**Figure S14**. Map of the urban‐rural transect for the city of Detroit.
**Figure S15**. Map of the urban‐rural transect for the city of Jacksonville.
**Figure S16**. Map of the urban‐rural transect for the city of Montreal.
**Figure S17**. Map of the urban‐rural transect for the city of New York.
**Figure S18**. Map of the urban‐rural transect for the city of Norfolk.
**Figure S19**. Map of the urban‐rural transect for the city of Philadelphia.
**Figure S20**. Map of the urban‐rural transect for the city of Pittsburgh.
**Figure S21**. Map of the urban‐rural transect for the city of Tampa.
**Figure S22**. Map of the urban‐rural transect for the city of Toronto.
**Figure S23**. Map of the urban‐rural transect for the city of Washington, D. C.
