# Supplementary material for: Predicting the strength of urban‐rural clines in a Mendelian polymorphism along a latitudinal gradient
Source: Evol Lett. 2020 Mar 25;4(3):212–25. doi: 10.1002/evl3.163 (PMC7293085; doi:10.1002/evl3.163)
Supplement: Supplementary file 1 — Table S1. Minimum and maximum years, and total number of weather observations for the months of January and February across all 16 cities. Table S2. Pearson product‐moment correlation coefficients (lower triangle) and associated P‐values (upper triangle) for all pairwise combinations of environmental variables collected for our analyses. Table S3. Best‐fit model for the change in within‐population HCN, Ac, or Li frequencies along our urbanization gradient. Table S4. Beta‐coefficients, standard errors, z‐statistics, and P‐values from binomial logistic regressions for each city using the individual plant phenotype data (i.e., HCN+ = 1; HCN− = 0) as the response variable, and standardized distance to the urban center as the sole predictor. Table S5. Top two models with ΔAICc < 2 returned from the model selection performed using ‘dredge’ in R. Table S6. “Full” model averaged coefficients from the model selection and averaging of top models with environmental predictors of mean HCN frequencies. Figure S1. Urban‐rural clines in the frequency of HCN within populations of Trifolium repens across 16 cities in eastern North America. Figure S2. Logistic clines fit to (a) Atlanta, (b) Baltimore, (c) Boston, and (d) Charlotte using the individual plant level phenotype data (i.e., HCN+ = 1; HCN− = 0) as the response variable (y‐axis) and standardized distance to the urban center as the sole predictor (x‐axis). Figure S3. Logistic clines fit to (a) Cincinnati, (b) Cleveland, (c) Detroit, and (d) Jacksonville using the individual plant level phenotype data (i.e., HCN+ = 1; HCN− = 0) as the response variable (y‐axis) and standardized distance to the urban center as the sole predictor (x‐axis). Figure S4. Logistic clines fit to (a) Montreal, (b) New York, (c) Norfolk, and (d) Philadelphia using the individual plant level phenotype data (i.e., HCN+ = 1; HCN− = 0) as the response variable (y‐axis) and standardized distance to the urban center as the sole predictor (x‐axis). Figure S5. [file EVL3-4-212-s001.docx]

**Online supplementary for: Predicting the strength of urban-rural clines in a Mendelian polymorphism along a latitudinal gradient**

**Authors:** James S. Santangelo^1, 2, 3*^, Ken A. Thompson^4^, Beata Cohan^1^, Jibran Syed^1^, Rob W. Ness^1, 2, 3^, and Marc T. J. Johnson^1, 2, 3^

**Contents:**

- Supplementary text:
  - Text S1: Detailed statistical analyses
  - Text S2: Power analyses for sampling design
  - Text S3: Assessing the fit of non-linear clines
  - Text S4: Analysis of herbivore damage
  - Text S5: Logistic regressions on individual plant data
- Supplementary tables: Tables S1 – S6
- Supplementary figures: Figures S1 – S23

**Supplementary text**

**Text S1: Detailed statistical analyses**

To assess the environmental predictors of HCN across our latitudinal gradient, we first calculated mean HCN frequencies for each region by averaging HCN frequencies across all urban and rural populations. We then fit a linear model with mean HCN frequency as our response variable and our environmental variables as predictors. However, because many environmental variables were highly correlated (Table S2), we reduced the total number of predictors included in our model using a two-step process similar to Kooyers and Olsen (2013). We first eliminated predictors that on their own did not significantly predict variation in HCN frequencies at *P* < 0.1, which resulted in the elimination of one variable (snow depth). We additionally removed mPET since it was highly correlated with, and is functionally the same as, aPET, and is incorporated into our alternative measure of aridity (i.e., SMD). Of the nine remaining environmental variables, we retained predictors as independent variables if they were not strongly correlated (|r| > 0.7) with any other predictor, which resulted in three variables (annual aridity, soil moisture deficit, and the # of days < 0°C with no snow cover) being retained. The remaining six variables were all strongly correlated (most |r| > 0.8), preventing us from being able to reliably interpret their independent effects at predicting HCN frequencies. We used principal components analysis (PCA) to summarize these six variables into a single new composite variable, PC1_HCN_. The PCA was performed on normally standardized environmental variables (i.e., mean of 0 and standard deviation of 1) using the ‘prcomp’ function from the ‘stats’ R package (R Core Team 2018). The first PC explained 90.2% of the variation in these variables (eigenvalue = 4.5). Because none of the other axes explained substantial variation (< 7%), we retained only the first PC axis. We refer to the first PC axis summarizing variation in the environmental variables retained for the analysis of HCN frequencies as PC1_HCN_. Lower values of PC1_HCN_ characterized cities with higher summer temperatures, higher minimum winter temperatures, higher summer precipitation, greater potential evapotranspiration, and lower snowfall. Our final model was as follows: HCN frequency ~ PC1_HCN_ + # days < 0°C with no snow + annual aridity index + soil moisture deficit. We fit this model using the ‘lm’ function in R and considered this the ‘full’ model, to which we performed model selection and averaging.

To examine which environmental factors best predict mean HCN frequencies, we generated reduced models with all pairwise combinations of predictors and ranked these models by AIC_c_ (Johnson and Omland 2004; Symonds and Moussalli 2011). To obtain parameter estimates and *P*-values, we averaged the model coefficients from all models with ΔAIC_c_ < 2 (Burnham and Anderson 2002; Richards 2005). Model selection and averaging was performed using the ‘dredge’ function in the ‘MuMIn’ R package (Bartoń 2016), which presents both ‘full’ and ‘conditional’ model coefficients. ‘Full’ model coefficients (not to be confused with the ‘full’ model generated before model selection) sets predictors to 0 if they are not included in component models whereas ‘conditional’ coefficients ignore component models where the predictor is absent. Thus, ‘full’ model coefficients are more conservative (Bartoń 2016) and we interpreted these to obtain parameter estimates and *P*-values.

Prior to examining the environmental predictors of clines in HCN and its component genes, we first tested whether, on average, urbanization influenced HCN frequencies. We fit a linear regression with the proportion of cyanogenic plants within each population as the response variable, and city, standardized distance to the urban center, and the city × distance interaction as predictors. We used distance to the urban center as a measure of urbanization as this is highly correlated with % impervious surface (R^2^ = 0.64, Johnson et al. 2018) and sufficiently captures variation in HCN frequencies across urban-rural gradients (Thompson et al. 2016; Johnson et al. 2018). Distances between populations were calculated using the haversine formula (Sinnott 1984). Because urban-rural transects varied in length, we standardized distance to be between 0 (urban-most) and 1 (rural-most). We fit this model using the ‘lm’ function in R and obtained *P*-values from type 3 sums-of-squares computed using the ‘Anova’ function in the R package ‘car’ (Fox and Weisberg 2011). In our model, a significant effect of City suggests that mean HCN frequencies vary across the 16 cities. A significant Distance term means that across all cities, HCN frequencies vary in parallel across the urban-rural transect (i.e., parallel clines in HCN frequencies), while a significant City × Distance interaction indicates the strength or direction of clines in HCN varies across cities. For the four cities for which we collected herbivore damage data (Detroit, Cincinnati, Cleveland, Pittsburgh), we fit models with the population-mean herbivore damage as the response variable and standardized distance to the urban center as the sole predictor. None of these cities showed significant changes in herbivore damage across the urbanization gradient (see supplementary text S5: “Analysis of herbivore damage”) so we do not present these results any further.

To examine the environmental predictors of variation in the strength of clines, we first fit separate linear models to the within-population HCN, *Ac*, or *Li* frequency data for each city. Each model contained the proportion of cyanogenic plants as the response variable and the standardized distance to the urban center as the predictor. Models were fit using the ‘lm’ function in R. To examine clines in the frequency of *Ac* and *Li*, we performed the same procedure as above but used the inferred frequency of *Ac* and *Li* from HWE as response variables. For each model, we extracted the beta coefficient (i.e., slope) describing the change in the frequency of HCN (or *Ac*/*Li*) per unit increase in the standardized distance to the urban center and used this as our estimate of the strength of the clines. Note that cities that showed significant changes in HCN frequency with distance (Table 1 in main text) were also significant following Bonferroni correction of logistic regressions using data from individual plants (i.e., 1 for HCN+, 0 for HCN−). Despite some cities being better fit by quadratic models (see supplementary text S3: “Assessing the fit of non-linear clines”, Table S3), we used the beta coefficient from a first-order linear regression for all cities to ensure that all slope values represented the linear change in the frequency of HCN with standardized distance to the urban center. We did not run a cline model for Tampa due to the absence of variation in HCN frequencies along the urban-rural transect (mean HCN frequency ~ 99%); thus, this analysis contains 15 cities.

We used the same procedure as above to reduce the number of environmental predictors of the strength of urban-rural clines in HCN. Only four of the 10 environmental variables (MST, MWT, snow depth, and snowfall) on their own significantly predicted the strength of clines in HCN and were retained for further analysis. However, because all of these predictors were highly correlated (all |r| > 0.86), we again used PCA to distill these variables into a smaller set of component axes. The first PC explained 92.8% of the variation in these four variables (eigenvalue = 3.7) while the remaining axes explained little variation (all variation explained < 4%). We thus retained only the loadings from this first PC in our model. We refer to the first PC axis summarizing variation in the environmental variables retained for the analysis of the strength of clines as PC1_Slope_. Cities with low values along PC1_slope_ have little snow and higher minimum winter and higher maximum summer temperatures. Since all environmental variables that predicted the strength of clines were incorporated into PC1_slope_, we did not perform model selection or averaging. Our final model included the strength of urban-rural clines in HCN as the response and PC1_slope_ as the sole predictor. The model was fit using the ‘lm’ function in R.

To examine whether urban and rural populations varied in the composition of deletion haplotypes, we first calculated to relative frequency of each haplotype at the *Ac* and *Li* loci for urban and rural populations for each of the seven cities for which haplotype data were available. To examine variation in deletion haplotypes across urban and rural habitats, we qualitatively examined the relative frequencies of deletion haplotypes in urban and rural habitats across the seven cities for which haplotype data were available. Finally, to examine variation in deletion haplotypes across urban and rural habitats, we used the raw counts of deletion haplotypes at each locus to calculate the haplotype richness (i.e., number of unique haplotype deletions) in urban and rural habitats for each city. We fit haplotype richness as the response variable in a linear model with habitat type (i.e., urban vs. rural) as the sole predictor such that a significant effect of habitat suggested differences in deletion haplotype richness among urban and rural habitats. All analyses were performed in R v. 3.6.1 (R Core Team 2019).

**Text S2: Power analyses for sampling design**

Because we intended to sample urban-rural transects from a large number of cities relative to previous work, we examined whether fewer plants per population could be sampled to save resources without compromising our ability to detect clines in HCN if they exist. To do this, we randomly resampled the data collected by Thompson *et al.* (2016) for the city of Boston. We chose to resample Boston as it showed the weakest cline in HCN, providing a conservative estimate of the number of plants necessary to reproduce urban-rural clines. We randomly resampling between 10 and 20 plants within populations, with replacement 5000 times, each time running a linear model with within-population HCN frequencies as the response variable and distance to urban center as the sole predictor. We then calculated the probability of observing a slope as large or larger than the one observed by Thompson *et al.* (2016) and examined the relative change in this probability for varying numbers of plants sampled, calculated as: [1 − P(X plants)] / P(20 Plants).

Decreasing the number of plants sampled within populations had little effect on the probability of detecting a clines as strong as the one observed in Boston by Thompson *et al.* (2016). Specifically, sampling 15 plants per population provided 99% of the statistical power as sampling 20 plants (i.e., only a 1% decrease in the probability of detecting a clines as strong as the one actually observed). Given this evidence of little change in statistical power, we chose to sample 15 plants from all populations along the urbanization gradients.

**Text S3: Assessing the fit of non-linear clines**

As described in text S1, we fit a linear model to each city with the mean within-population HCN frequency (or *Ac* and *Li* frequencies, where available) as a response variable and standardized distance to the urban center as a predictor. However, visual inspection of some of the regression plots suggested that some cities may be better fit by quadratic models rather than first-order regressions. Therefore, for each regression in each city, we additionally fit quadratic models to the within-population HCN, *Ac*, or *Li*, frequencies (i.e., Response ~ std_distance + std_distance^2^). We considered the quadratic model a better fit if it was more that 2 AIC points lower than the AIC score for the first-order regression.

While most cities were best fit by first-order regressions for HCN, *Ac*, and *Li*, some cities were better fit by quadratic cline models (Table S3). In particular, both Charlotte and Jacksonville showed non-linear changes in the frequency of HCN with increasing distance from the urban center (Table S3, Figure S8 and S12). While no cities showed non-linear changes in the frequency of *Ac* with increasing distance from the urban center, Atlanta (Figure S5), Charlotte (Figure S8), Jacksonville (Figure S12) and Washington (Figure S20) all varied non-linearly in the predicted frequency of *Li* along our urbanization gradient (Table S3).

**Text S4: Analysis of herbivore damage**

Herbivory did not vary with urbanization in any of the four cities in which we tested for this. This included Cincinnati (*β* = −0.024 ± 0.034 SE, *P* = 0.50), Cleveland (*β* = −0.028 ± 0.029 SE, *P* = 0.25), Detroit (*β* = 0.010 ± 0.026 SE, *P* = 0.71), or Pittsburgh (*β* = −0.014 ± 0.044 SE, *P* = 0.75) showed significant change in herbivore pressure across the urbanization gradient. This suggests that urbanization is not driving changes in herbivore pressure on white clover plants in these four northern cities.

**Text S5: Logistic regressions on individual plant data**

In addition to our analyses examining variation in population-mean HCN frequency with urbanization and the environmental predictors of the strength of clines, we performed logistic regressions on the raw individual plant phenotype data. We began by fitting a binomial logistic regression using the raw plant-level HCN data (HCN+ = 1; HCN− = 0) as the response variable and standardized distance, city, and the city × distance interaction as fixed effect predictors. We obtained parameter estimates and P-values using type III SS and likelihood ratio tests of individual model predictors.

We additionally fit a binomial logistic regression for each city individually using the raw plant-level HCN data (HCN+ = 1; HCN− = 0) as the response variable and standardized distance to the urban center as the sole predictor (table S4). For each regression, we extracted the beta-coefficient representing the change in the log-odds of being cyanogenic per unit increase in the standardized distance from the city centre and used this as our measure for the strength of clines. We then performed the same analysis described in the main text and in text S1 to identify the environmental predictors of variation in the strength of clines across cities (i.e., PCA of environmental variables followed by regression using PC1 as a predictor). Similar to PC1_slope_ in the main text, PC1_slopeLog_ in this case explained 87% of the variation in annual PET, monthly precipitation, minimum winter temperature, maximum summer temperature, snow depth, and snowfall. Cities with low values along PC1_slopeLog_ get little snow, have high summer and winter temperatures, and high precipitation and annual potential evapotranspiration, while cities with high values along PC1_slopeLog_ have the opposite.

Averaged across cities, the predicted probability of being cyanogenic increased from 26% in the urban-most population to 42% in the rural-most population, although this effect was not significant in the type III model (*β* ± SE= 0.24 ± 0.30, 𝛸^2^_1_ = 0.88, *P* = 0.35, fig. S2). The strength of clines varied among cities (standardized distance × city effect, 𝛸^2^_15_ = 104.09, *P* < 0.001, Table S4, fig. S2 – S6), justifying further examination into the environmental predictors of variation in the strength of cyanogenesis clines among cities. The strength of urban-rural clines—measured here as the change in the log-odds that a plant is cyanogenic with increasing distance—decreased with increasing values along PC1_SlopeLog_ (*β* = −0.222 ± 0.08 SE, *t*_13_ = −2.27, *P* = 0.01, R^2^ = 38%, fig. S7), again implying that the strongest clines occurred in the warmest environments and the weakest clines occurred in regions of low temperature and high snowfall/depth.

**Supplementary tables**

**Table S1**: Minimum and maximum years, and total number of weather observations for the months of January and February across all 16 cities. Weather data were obtained as daily values from a single international airport in each city.

| City | Airport station | min_year | max_year | count |
| --- | --- | --- | --- | --- |
| Atlanta | Hartsfield International | 1980 | 2015 | 1612 |
| Baltimore | Washington International | 1980 | 2015 | 2133 |
| Boston | Logan International | 1980 | 2004 | 1364 |
| Charlotte | Douglas International | 1980 | 2015 | 2133 |
| Cincinnati | Northern Kentucky International | 1980 | 2015 | 2133 |
| Cleveland | Hopkins International | 1980 | 2015 | 2132 |
| Detroit | Detroit Metropolitan | 1980 | 2015 | 2122 |
| Jacksonville | Jacksonville International | 1980 | 2015 | 1538 |
| Montreal | Pierre Elliott Trudeau International | 1980 | 2015 | 2109 |
| NewYork | La Guardia | 1980 | 2015 | 2133 |
| Norfolk | Norfolk International | 1980 | 2015 | 1120 |
| Philadelphia | Philadelphia International | 1980 | 2015 | 2073 |
| Pittsburgh | Pittsburgh International | 1980 | 2015 | 2132 |
| Tampa | Tampa International | 1980 | 2015 | 2133 |
| Toronto | Lester B. Pearson International | 1980 | 2013 | 2006 |
| Washington D.C. | Dc Dulles International | 1980 | 2015 | 2132 |

**Table S2**: Pearson product-moment correlation coefficients (lower triangle) and associated *P*-values (upper triangle) for all pairwise combinations of environmental variables collected for our analyses. Bolded coefficients and *P-*values are significant at *P* < 0.05.

|  | **Lat** | **Long** | **AI** | **mPET** | **aPET** | **mPrecip** | **MWT** | **MST** | **SMD** | **SD** | **SF** | **Days < 0°C no snow** |
| --- | --- | --- | --- | --- | --- | --- | --- | --- | --- | --- | --- | --- |
| **Lat** |  | 0.058 | **0.042** | **< 0.001** | **< 0.001** | **< 0.001** | **< 0.001** | **< 0.001** | **0.035** | **< 0.001** | **< 0.001** | 0.263 |
| **Long** | 0.484 |  | **0.001** | **0.041** | 0.062 | 0.397 | 0.155 | 0.21 | 0.808 | 0.215 | 0.171 | 0.42 |
| **AI** | **0.513** | **0.751** |  | **0.021** | **0.032** | 0.252 | 0.078 | 0.116 | 0.987 | 0.181 | 0.098 | 0.568 |
| **mPET** | **-0.876** | **-0.516** | **-0.57** |  | **< 0.001** | **0.007** | **< 0.001** | **< 0.001** | 0.618 | **< 0.001** | **< 0.001** | 0.891 |
| **aPET** | **-0.986** | -0.477 | **-0.538** | **0.938** |  | **< 0.001** | **< 0.001** | **< 0.001** | 0.097 | **< 0.001** | **< 0.001** | 0.415 |
| **mPrecip** | **-0.882** | -0.227 | -0.304 | **0.644** | **0.837** |  | **< 0.001** | **< 0.001** | **< 0.001** | **0.014** | **0.003** | 0.06 |
| **MWT** | **-0.983** | -0.373 | -0.453 | **0.806** | **0.953** | **0.905** |  | **< 0.001** | **0.012** | **< 0.001** | **< 0.001** | 0.284 |
| **MST** | **-0.938** | -0.331 | **-0.409** | **0.893** | **0.95** | **0.775** | **0.93** |  | 0.147 | **< 0.001** | **< 0.001** | 0.859 |
| **SMD** | **-0.529** | 0.066 | 0.004 | 0.135 | 0.429 | **0.845** | **0.609** | 0.38 |  | 0.45 | 0.222 | **0.007** |
| **SD** | **0.817** | 0.328 | 0.352 | **-0.821** | **-0.821** | **-0.6** | **-0.82** | **-0.888** | -0.203 |  | **< 0.001** | 0.34 |
| **SF** | **0.896** | 0.36 | 0.429 | **-0.824** | **-0.891** | **-0.694** | **-0.886** | **-0.941** | -0.323 | **0.91** |  | 0.88 |
| **Days < 0°C no snow** | 0.298 | 0.217 | 0.154 | 0.037 | -0.219 | -0.48 | -0.286 | -0.048 | **-0.648** | -0.255 | 0.041 |  |

Abbreviations: Latitude (Lat); longitude (Long); annual aridity index (AI); monthly PET (mPET); annual PET (aPet); monthly precipitation (mPrecip); minimum winter temperature (MWT); maximum summer temperature (MST); soil moisture deficit (SMD); snow depth (SD); snowfall (SF).

**Table S3:** Best-fit model for the change in within-population HCN, *Ac*, or *Li* frequencies along our urbanization gradient. “Linear” implies that HCN, *Ac*, or *Li* frequencies changed in a linear fashion along our urbanization gradient, whereas “quadratic” means the change in the frequency of HCN, *Ac*, or *Li* was non-linear along our transect. ‘NS’ represents non-significant changes in HCN, *Ac*, or *Li* frequencies along our urbanization gradient. Grey cells show cities for which data at the individual loci was not collected.

| **City** | **HCN** | ***Ac*** | ***Li*** |
| --- | --- | --- | --- |
| Atlanta | linear | linear | quadratic |
| Baltimore | NS | NS | NS |
| Boston | linear |  |  |
| Charlotte | quadratic | NS | quadratic |
| Cincinnati | NS |  |  |
| Cleveland | NS | NS | NS |
| Detroit | NS |  |  |
| Jacksonville | quadratic | linear | quadratic |
| Montreal | NS |  |  |
| New York | linear | linear | NS |
| Norfolk | linear | linear | NS |
| Philadelphia | NS |  |  |
| Pittsburgh | NS |  |  |
| Tampa | NS |  |  |
| Toronto | linear | linear | linear |
| Washington, D.C. | linear | linear | quadratic |

**Table S4:** Beta-coefficients, standard errors, z-statistics, and *P*-values from binomial logistic regressions for each city using the individual plant phenotype data (i.e., HCN+ = 1; HCN− = 0) as the response variable, and standardized distance to the urban center as the sole predictor.

| **City** | ***β*** | **SE** | **𝑧** | ***P*** |
| --- | --- | --- | --- | --- |
| **Atlanta** | **1.467** | **0.252** | **5.823** | **< 0.001** |
| Baltimore | 0.184 | 0.309 | 0.596 | 0.551 |
| **Boston** | **0.755** | **0.277** | **2.728** | **0.006** |
| Charlotte | 0.258 | 0.254 | 1.016 | 0.310 |
| Cincinnati | 0.235 | 0.321 | 0.734 | 0.463 |
| Cleveland | 0.469 | 0.295 | 1.592 | 0.111 |
| Detroit | 0.237 | 0.275 | 0.862 | 0.389 |
| **Jacksonville** | **2.978** | **0.509** | **5.855** | **< 0.001** |
| Montreal | -0.220 | 0.223 | -0.987 | 0.324 |
| **NewYork** | **0.990** | **0.277** | **3.576** | **< 0.001** |
| **Norfolk** | **1.446** | **0.259** | **5.591** | **< 0.001** |
| Philadelphia | -0.212 | 0.329 | -0.645 | 0.519 |
| Pittsburgh | 0.378 | 0.300 | 1.259 | 0.208 |
| Tampa | -5.780 | 4.639 | -1.246 | 0.213 |
| **Toronto** | **1.311** | **0.144** | **9.103** | **< 0.001** |
| **Washington D.C.** | **0.956** | **0.262** | **3.643** | **< 0.001** |

**Table S5**: Top two models with ΔAIC_c_ < 2 returned from the model selection performed using ‘dredge’ in R. Shown are the *β* coefficients for terms included in the model, the model R^2^, *F*-statistic, degrees-of-freedom (df), log-likelihood (LL), AIC_c_, ΔAIC_c_ and model weight. Grey cells represent terms not included in the specific model shown. These two models were averaged to produce the “conditional” and “full” model averaged coefficients in Tables S5 and S6, respectively.

|  | **Estimate (*β*)** | | | | |  |  |  |  |  |  |  |
| --- | --- | --- | --- | --- | --- | --- | --- | --- | --- | --- | --- | --- |
| **Model** | **Intercept** | **AI** | **# Days < 0°C no snow** | **PC1_HCN_** | **SMD** | **R^2^** | **F** | **df** | **LL** | **AIC_c_** | **ΔAIC_c_** | **weight** |
| 1 | 0.98 | −0.29 | −0.015 | −0.06 |  | 0.96 | 89.28 | 5 | 25.88 | −35.76 | 0.000 | 0.53 |
| 2 | 0.69 |  | −0.015 | −0.07 |  | 0.94 | 104.26 | 4 | 23.37 | −35.11 | 0.66 | 0.38 |

Abbreviations: annual aridity index (AI); soil moisture deficit (SMD)

**Table S6:** “Full” model averaged coefficients from the model selection and averaging of top models with environmental predictors of mean HCN frequencies. Top two models were within 2 AIC_c_ points of one another (Table S4).

| **Term** | ***β*** | **SE** | **𝑧** | ***P*** |
| --- | --- | --- | --- | --- |
| **Annual aridity** | -0.170 | 0.179 | 0.912 | 0.362 |
| **# days < 0 °C no snow** | **-0.015** | **0.002** | **8.774** | **< 0.001** |
| **PC1_HCN_** | **-0.064** | **0.008** | **6.987** | **< 0.001** |

**Supplementary figures**

**
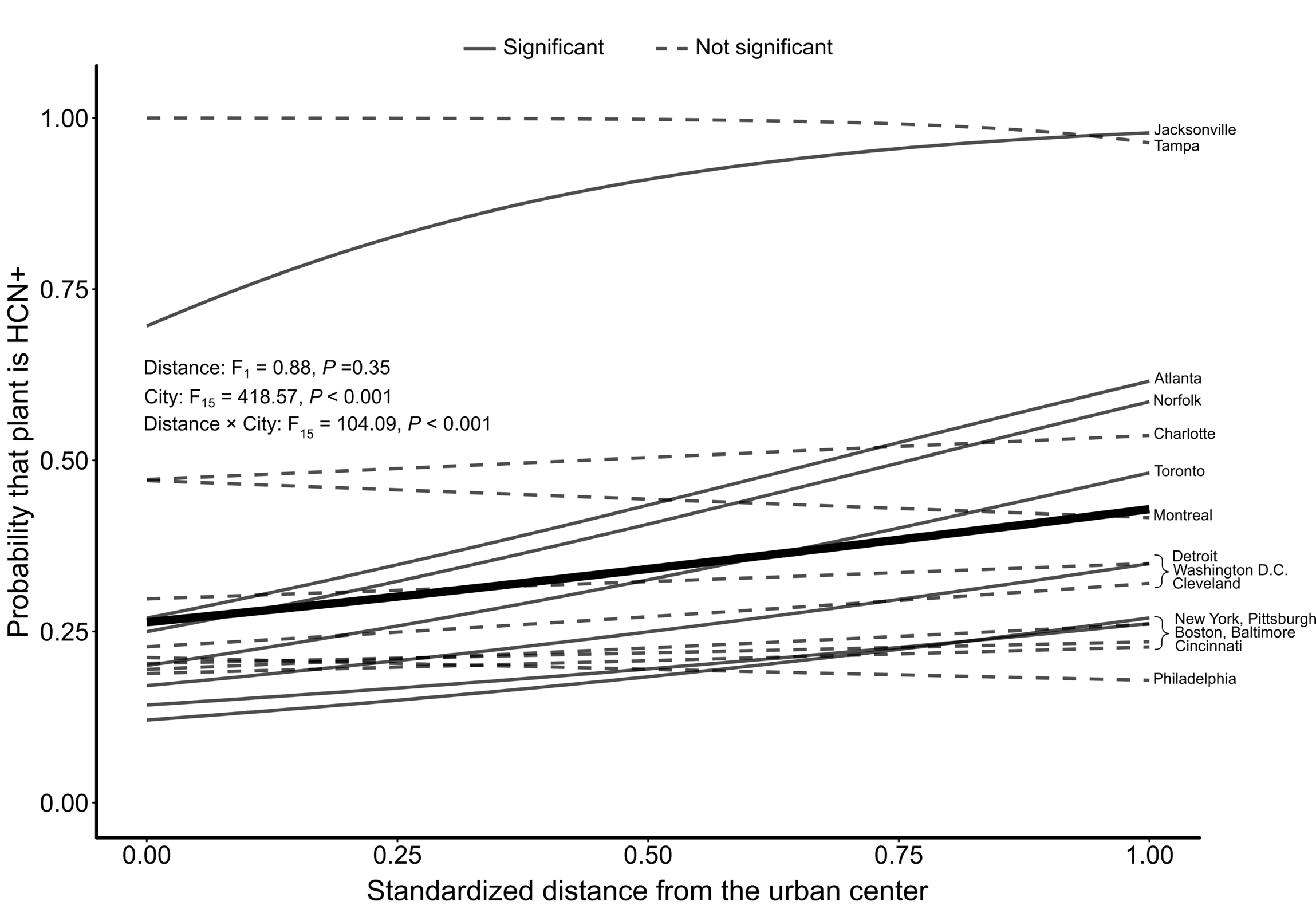
**

**Figure S1:** Urban-rural clines in the frequency of HCN within populations of *Trifolium repens* across 16 cities in eastern North America. The probability that a *T. repens* individual is cyanogenic is plotted against the standardized distance from the urban center. Solid lines represent linear regressions from cities where the logistic regression showed a significant cline in HCN at *P* < 0.05, whereas dashed lines are cities that lack significant clinal variation. The thick black line represents the main effect of standardized distance on HCN frequencies, averaged across all cities.

**Figure S2:** Logistic clines fit to (a) Atlanta, (b) Baltimore, (c) Boston, and (d) Charlotte using the individual plant level phenotype data (i.e., HCN+ = 1; HCN− = 0) as the response variable (y-axis) and standardized distance to the urban center as the sole predictor (x-axis). Grey shading around sigmoid curve represent the 95% confidence interval of the fitted line.

**Figure S3:** Logistic clines fit to (a) Cincinnati, (b) Cleveland, (c) Detroit, and (d) Jacksonville using the individual plant level phenotype data (i.e., HCN+ = 1; HCN− = 0) as the response variable (y-axis) and standardized distance to the urban center as the sole predictor (x-axis). Grey shading around sigmoid curve represent the 95% confidence interval of the fitted line.

**Figure S4:** Logistic clines fit to (a) Montreal, (b) New York, (c) Norfolk, and (d) Philadelphia using the individual plant level phenotype data (i.e., HCN+ = 1; HCN− = 0) as the response variable (y-axis) and standardized distance to the urban center as the sole predictor (x-axis). Grey shading around sigmoid curve represent the 95% confidence interval of the fitted line.

**Figure S5:** Logistic clines fit to (a) Pittsburgh, (b) Toronto, and (c) Washingtom D. C. using the individual plant level phenotype data (i.e., HCN+ = 1; HCN− = 0) as the response variable (y-axis) and standardized distance to the urban center as the sole predictor (x-axis). Grey shading around sigmoid curve represent the 95% confidence interval of the fitted line.

Figure S6: The strength of urban-rural clines in HCN—measured here as the change in the log-odds that a plant is cyanogenic with increasing distance to the urban center (y-axis)—was influenced by PC1_SlopeLog_, a composite axis that accounts for 87% of the variation in annual PET, monthly precipitation, minimum winter temperature, maximum summer temperature, snow depth, and snowfall. Cities with low values along PC1_slopeLog_ get little snow, have high summer and winter temperatures, and high precipitation and annual potential evapotranspiration, while cities with high values along PC1_slopeLog_ have the opposite. City labels are slightly jittered to avoid overlap. Bolded cities shower significant linear changes in HCN along urbanization gradients. City abbreviations: Jacksonville (Jax); Tampa (Tpa); Atlanta (Atl); Norfolk (Nor); Charlotte (Clt); Toronto (Tor); Montréal (Mtl); Detroit (Det); Washington D.C. (DC); Cleveland (Clv); New York (NY); Pittsburgh (Pgh); Boston (Bos); Baltimore (Blt); Cincinnati (Cin); Philadelphia (Phl).

**Figure S7:** Mean frequency of HCN within a city as a function of latitude. HCN frequency is highest in more southern and norther populations. The quadratic model provided a significantly better fit than the linear model based on AIC_c_ (AIC_c_linear_ = −8.8, AIC_c_quadratic_ = −32.8).

**
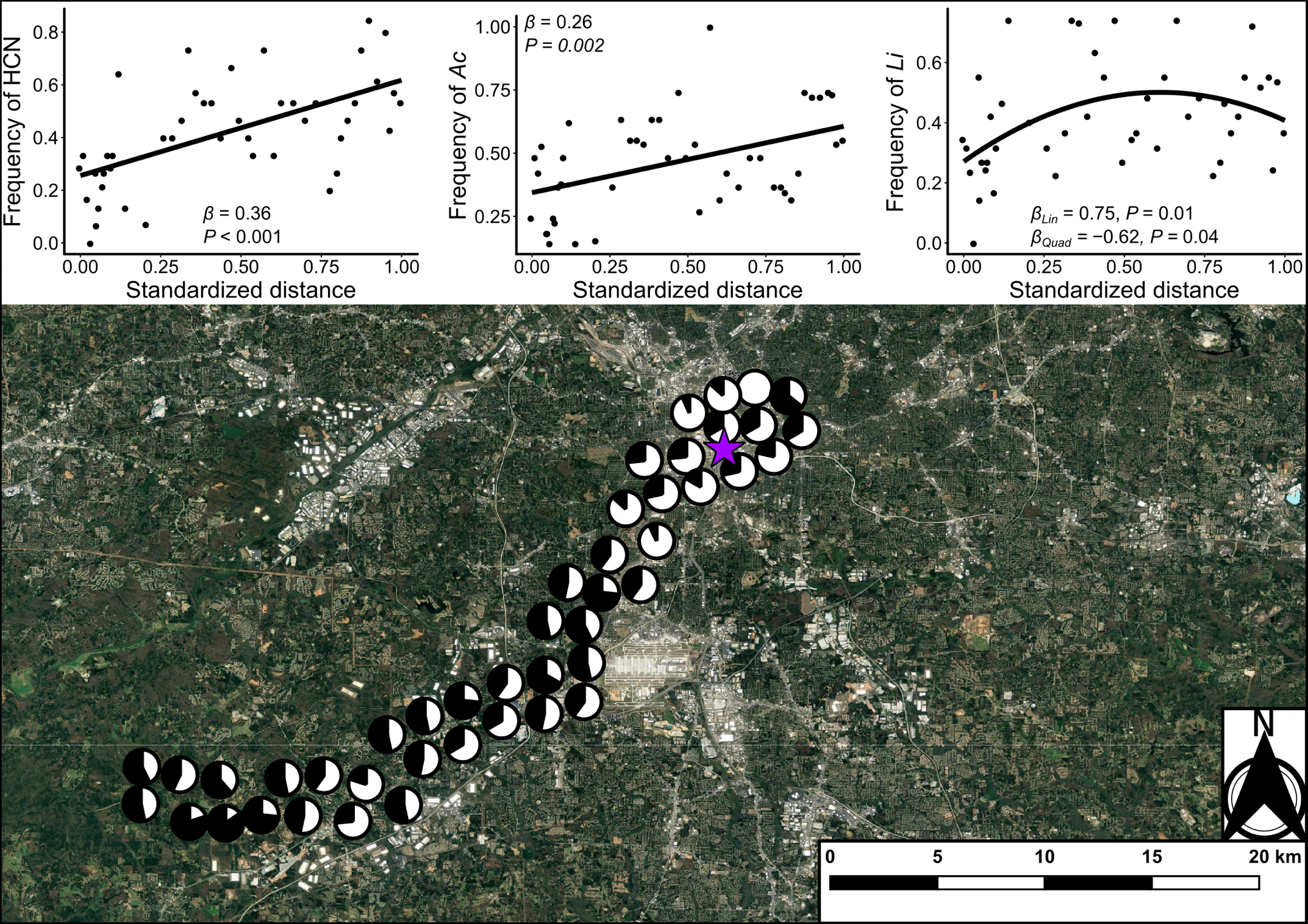
**

**Figure S8**: Map of the urban-rural transect for the city of Atlanta. Populations along the transect are represented with pie charts showing the proportion of cyanogenic plants (black) in the population. Pie charts have been jittered from their actual location to improve visualization. The purple star represents the location of the city center (Lat: 33.748997, Long: −84.387985). Inset shows the best fit regressions for the change in the frequency of HCN, *Ac*, and *Li* along an urbanization gradient, using standardized distance to the city center as a predictor. For each cline, slopes (*β*) and *P*-values for first-order (linear) and second-order (quadratic, where applicable) terms are provided.


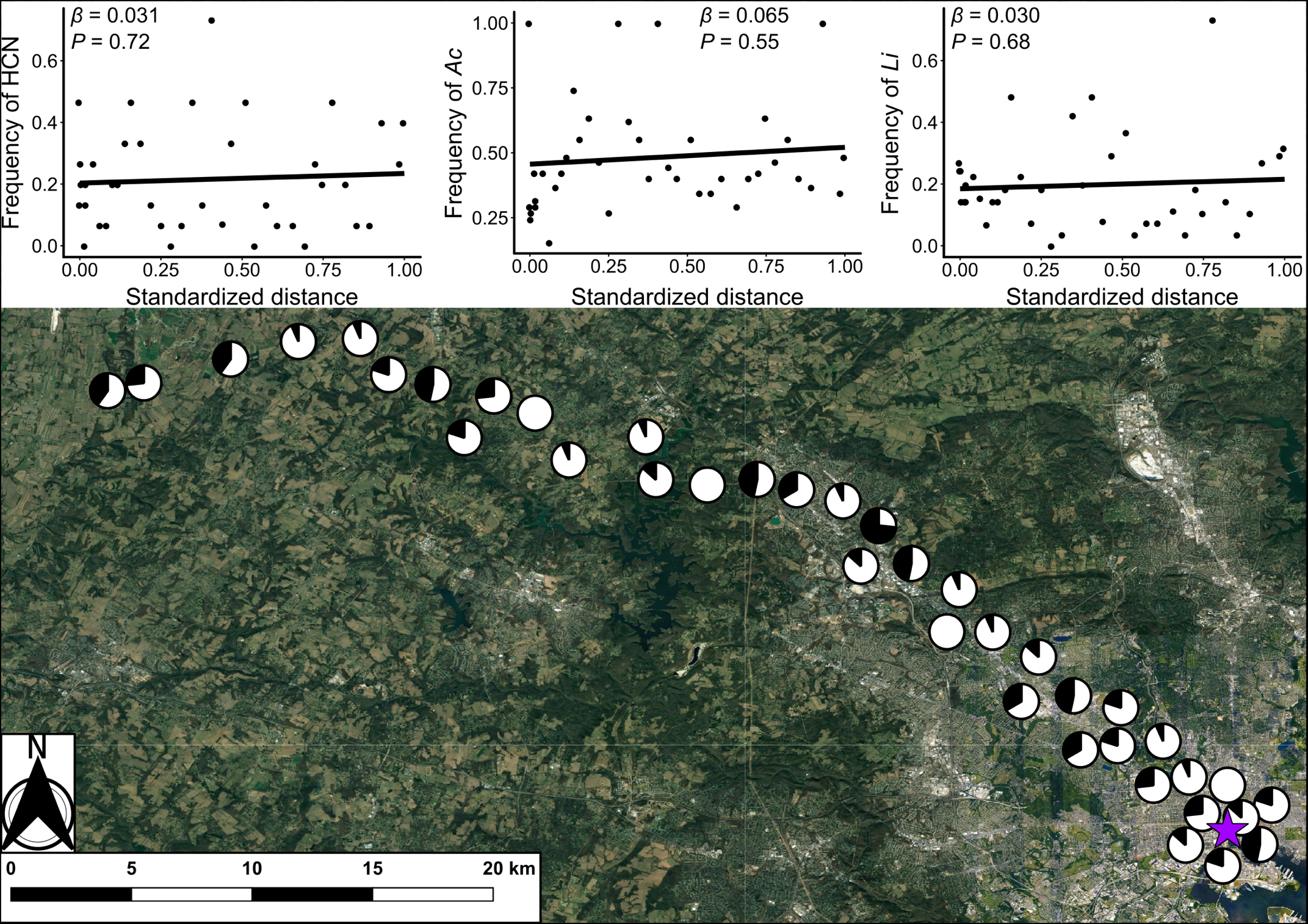


**Figure S9**: Map of the urban-rural transect for the city of Baltimore. Populations along the transect are represented with pie charts showing the proportion of cyanogenic plants (black) in the population. Pie charts have been jittered from their actual location to improve visualization. The purple star represents the location of the city center (Lat: 39.29039, Long: −76.61219). Inset shows the best fit regressions for the change in the frequency of HCN, *Ac*, and *Li* along an urbanization gradient, using standardized distance to the city center as a predictor. For each cline, slopes (*β*) and *P*-values for first-order (linear) terms are provided.


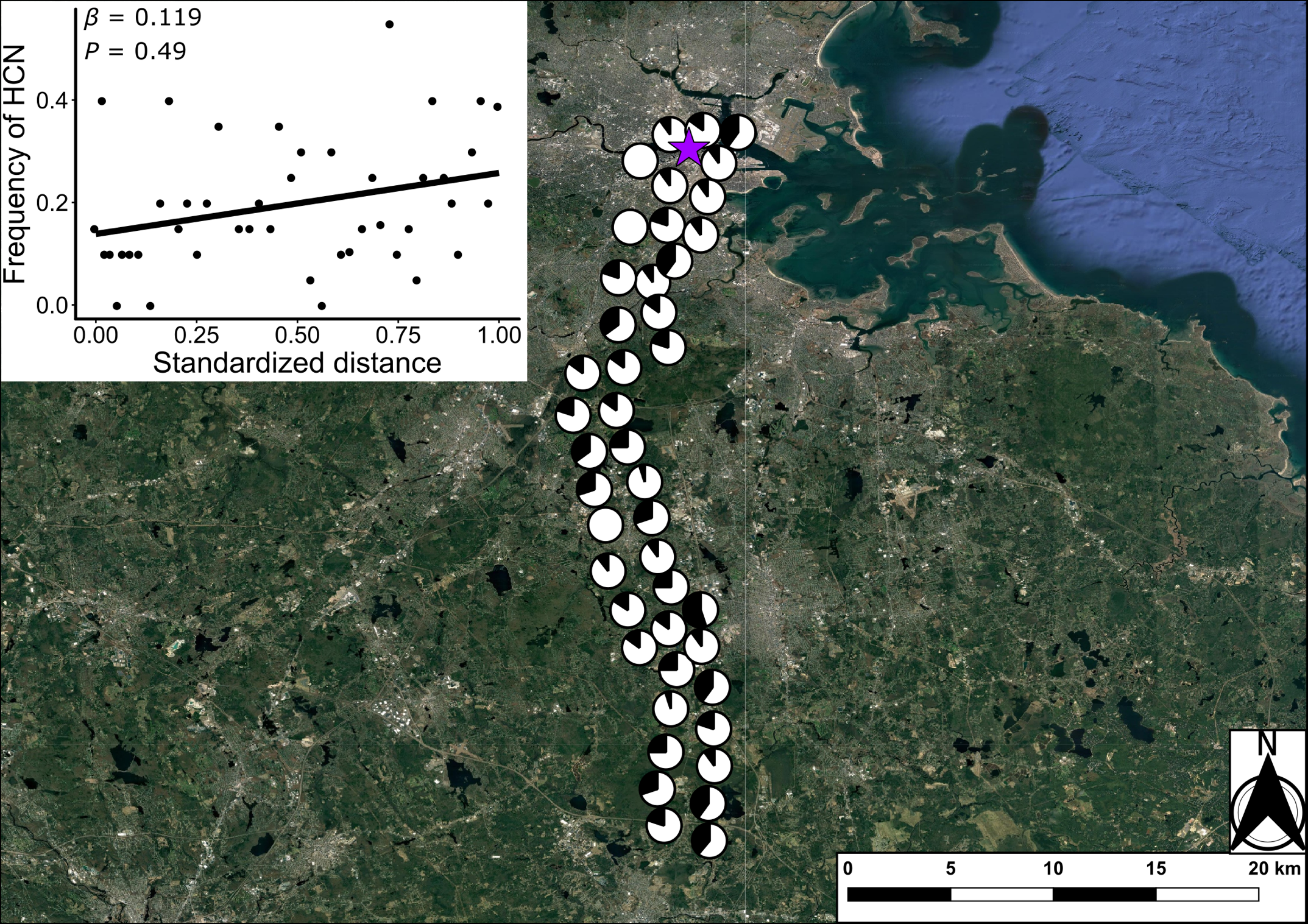


**Figure S10**: Map of the urban-rural transect for the city of Boston. Populations along the transect are represented with pie charts showing the proportion of cyanogenic plants (black) in the population. Pie charts have been jittered from their actual location to improve visualization. The purple star represents the location of the city center (Lat: 42.3547, Long: −71.0665). Inset shows the best fit regressions for the change in the frequency of HCN along an urbanization gradient, using standardized distance to the city center as a predictor. The slope (*β*) and *P*-value for first-order (linear) regression is provided.


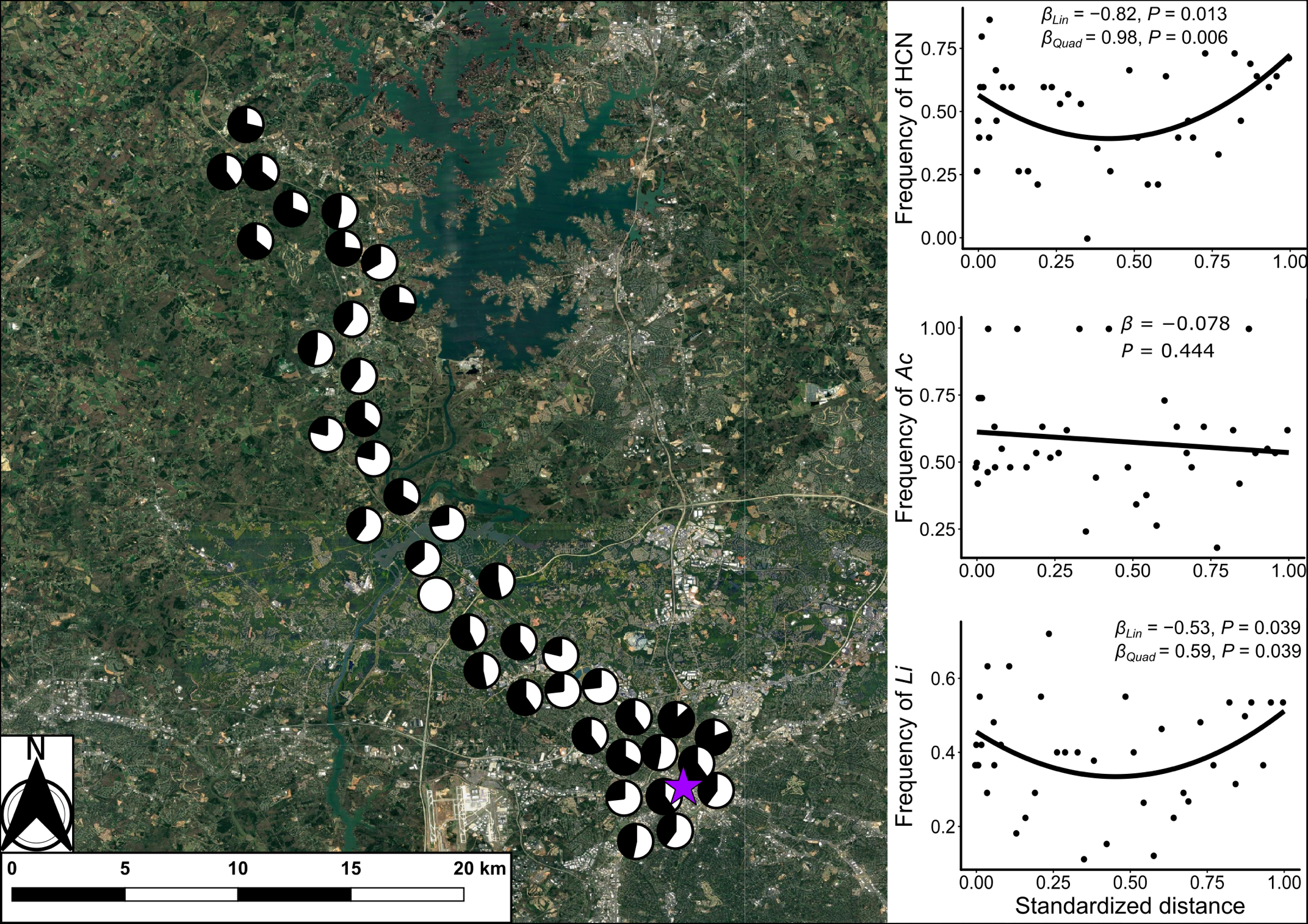


**Figure S11**: Map of the urban-rural transect for the city of Charlotte. Populations along the transect are represented with pie charts showing the proportion of cyanogenic plants (black) in the population. Pie charts have been jittered from their actual location to improve visualization. The purple star represents the location of the city center (Lat: 35.227085, Long: −80.843124). Inset shows the best fit regressions for the change in the frequency of HCN, *Ac*, and *Li* along an urbanization gradient, using standardized distance to the city center as a predictor. For each cline, slopes (*β*) and *P*-values for first-order (linear) and second-order (quadratic, where applicable) terms are provided.


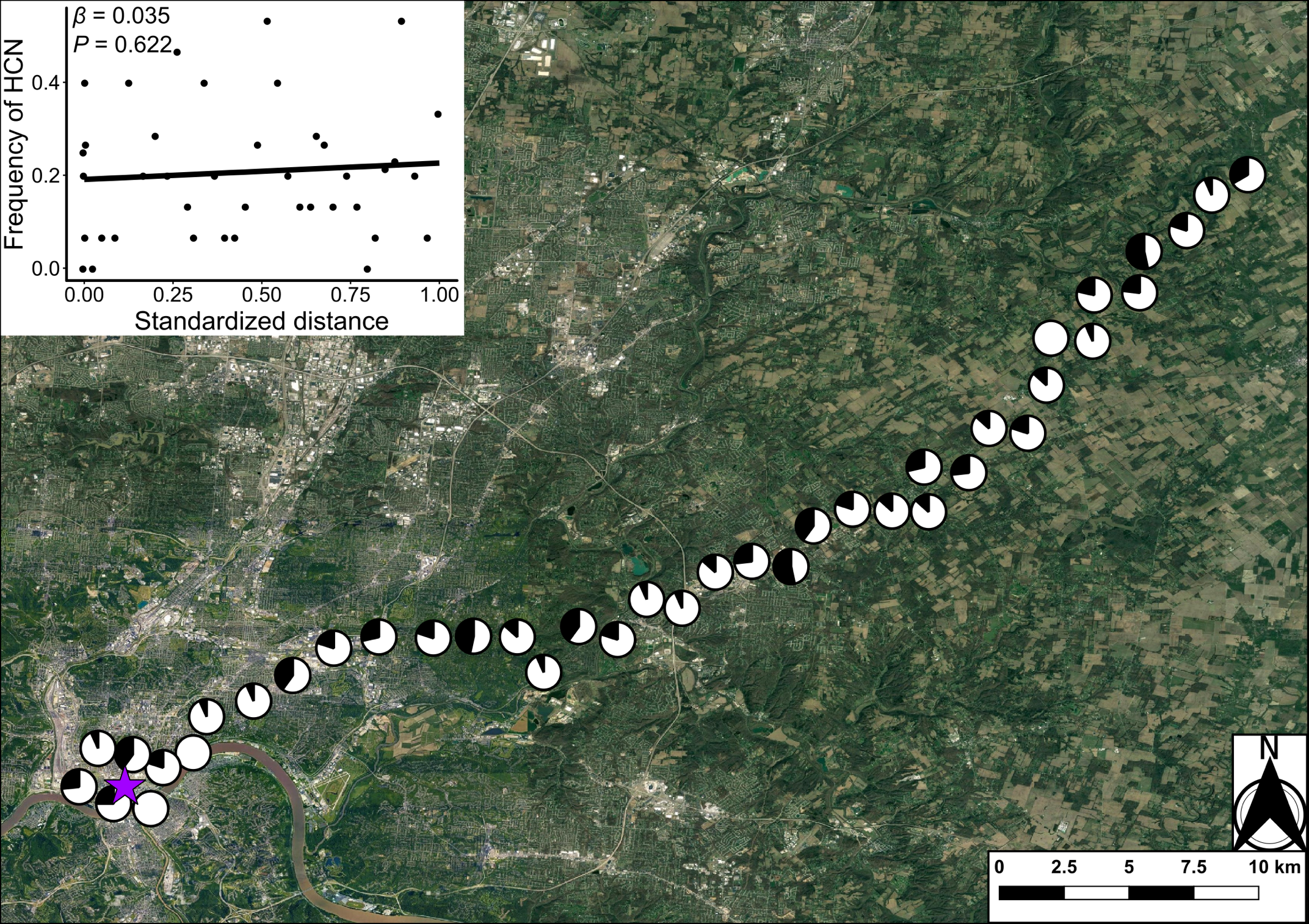


**Figure S12**: Map of the urban-rural transect for the city of Cincinnati. Populations along the transect are represented with pie charts showing the proportion of cyanogenic plants (black) in the population. Pie charts have been jittered from their actual location to improve visualization. The purple star represents the location of the city center (Lat: 39.103119, Long: −84.512016). Inset shows the best fit regressions for the change in the frequency of HCN along an urbanization gradient, using standardized distance to the city center as a predictor. The slope (*β*) and *P*-value for first-order (linear) regression is provided.


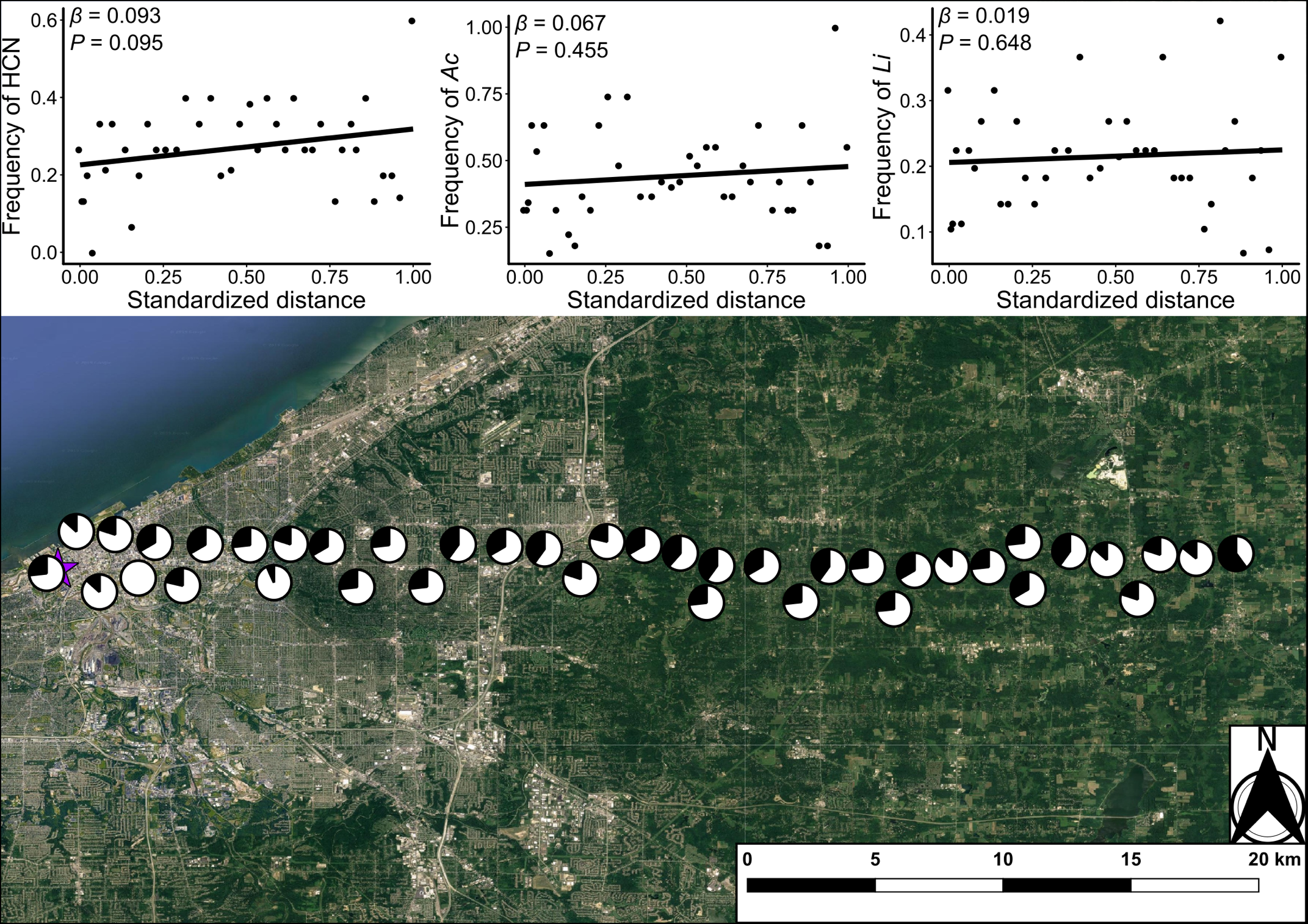


**Figure S13**: Map of the urban-rural transect for the city of Cleveland. Populations along the transect are represented with pie charts showing the proportion of cyanogenic plants (black) in the population. Pie charts have been jittered from their actual location to improve visualization. The purple star represents the location of the city center (Lat: 41.499321, Long: −81.694359). Inset shows the best fit regressions for the change in the frequency of HCN, *Ac*, and *Li* along an urbanization gradient, using standardized distance to the city center as a predictor. For each cline, slopes (*β*) and *P*-values for first-order (linear) terms are provided.


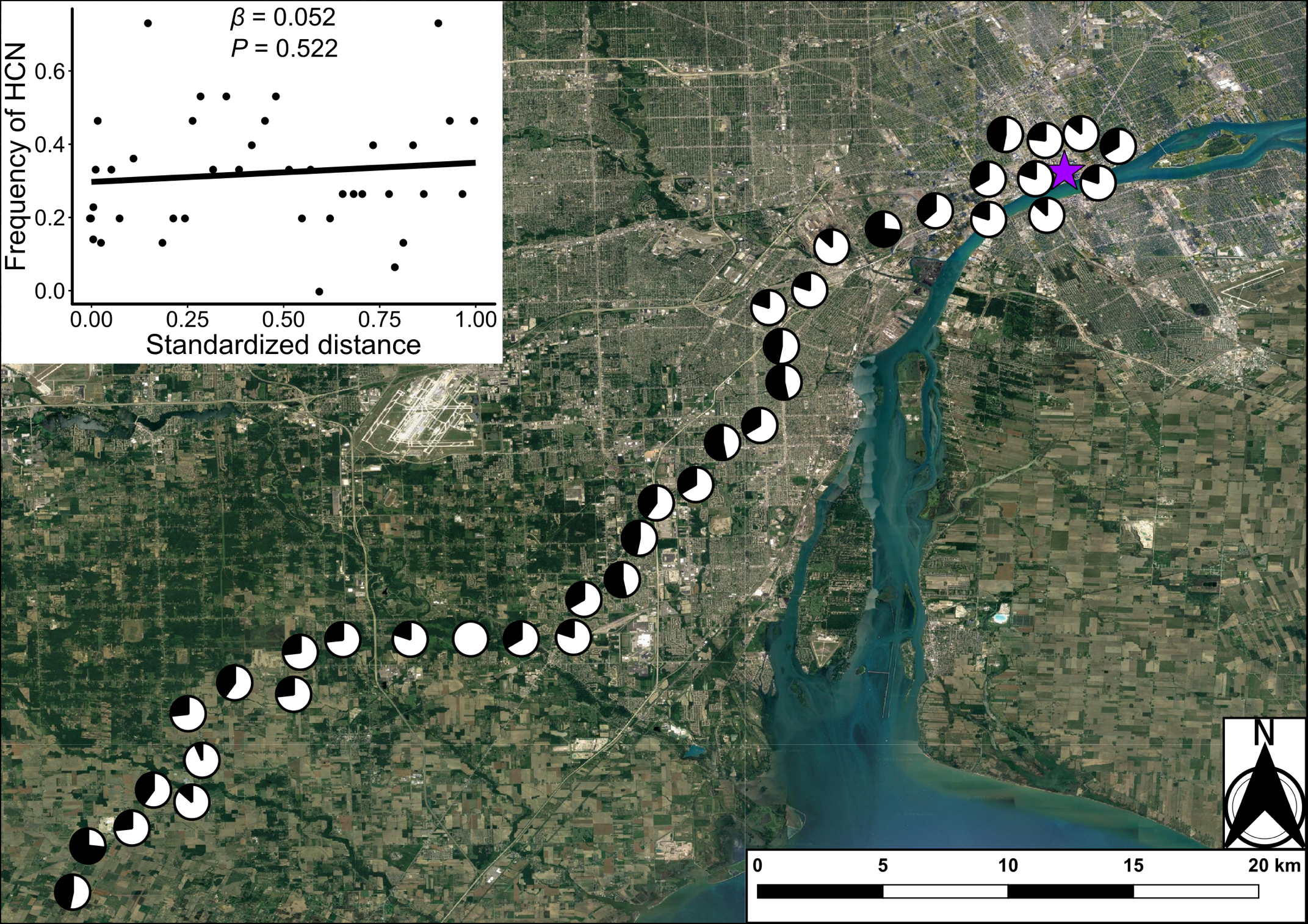


**Figure S14**: Map of the urban-rural transect for the city of Detroit. Populations along the transect are represented with pie charts showing the proportion of cyanogenic plants (black) in the population. Pie charts have been jittered from their actual location to improve visualization. The purple star represents the location of the city center (Lat: 42.331429, Long: −83.045753). Inset shows the best fit regressions for the change in the frequency of HCN along an urbanization gradient, using standardized distance to the city center as a predictor. The slope (*β*) and *P*-value for first-order (linear) regression is provided.


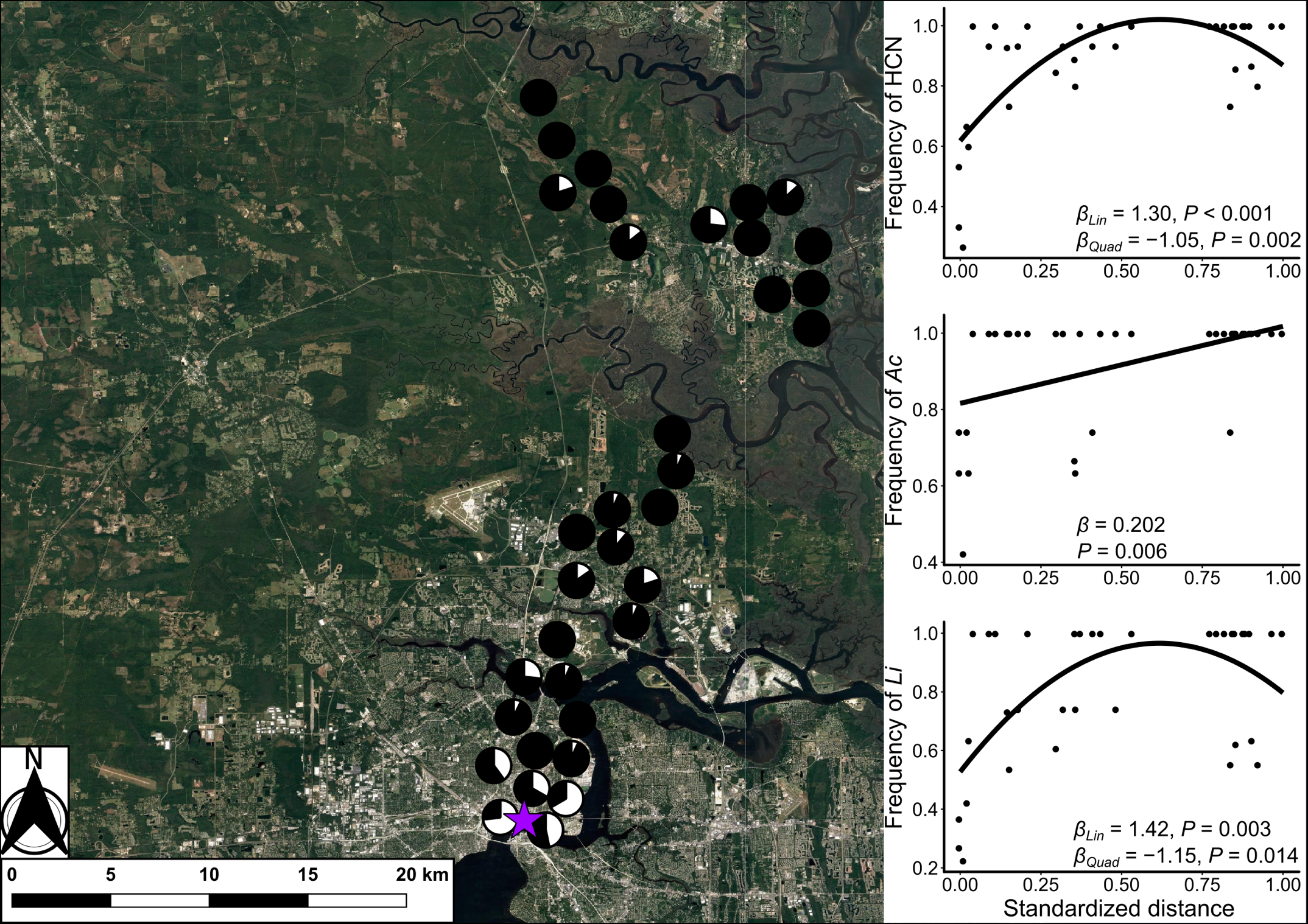


**Figure S15**: Map of the urban-rural transect for the city of Jacksonville. Populations along the transect are represented with pie charts showing the proportion of cyanogenic plants (black) in the population. Pie charts have been jittered from their actual location to improve visualization. The purple star represents the location of the city center (Lat: 30.32597, Long: − 81.656761). Inset shows the best fit regressions for the change in the frequency of HCN, *Ac*, and *Li* along an urbanization gradient, using standardized distance to the city center as a predictor. For each cline, slopes (*β*) and *P*-values for first-order (linear) and second-order (quadratic, where applicable) terms are provided.


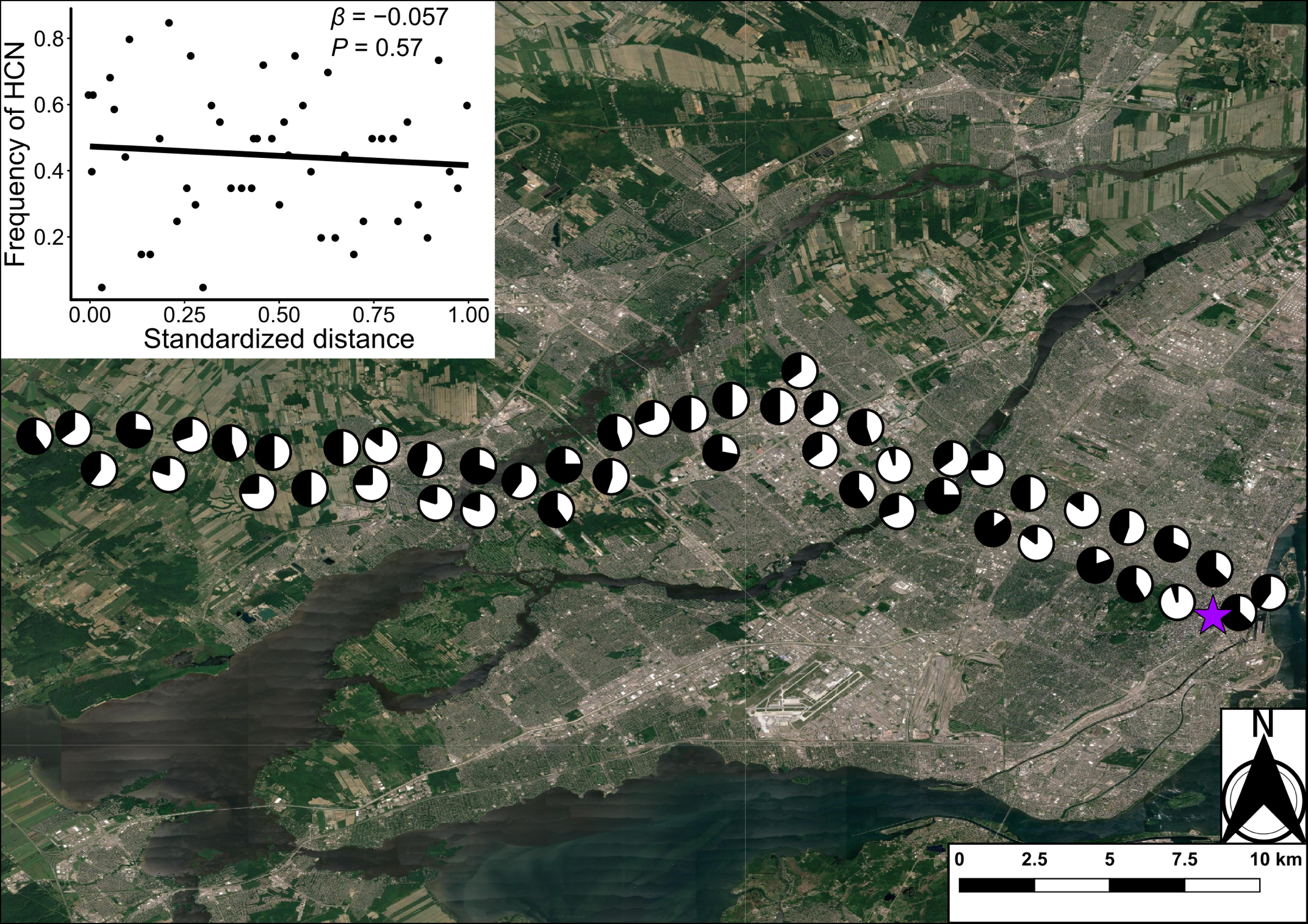


**Figure S16**: Map of the urban-rural transect for the city of Montreal. Populations along the transect are represented with pie charts showing the proportion of cyanogenic plants (black) in the population. Pie charts have been jittered from their actual location to improve visualization. The purple star represents the location of the city center (Lat: 45.502, Long: −73.5672). Inset shows the best fit regressions for the change in the frequency of HCN along an urbanization gradient, using standardized distance to the city center as a predictor. The slope (*β*) and *P*-value for first-order (linear) regression is provided.


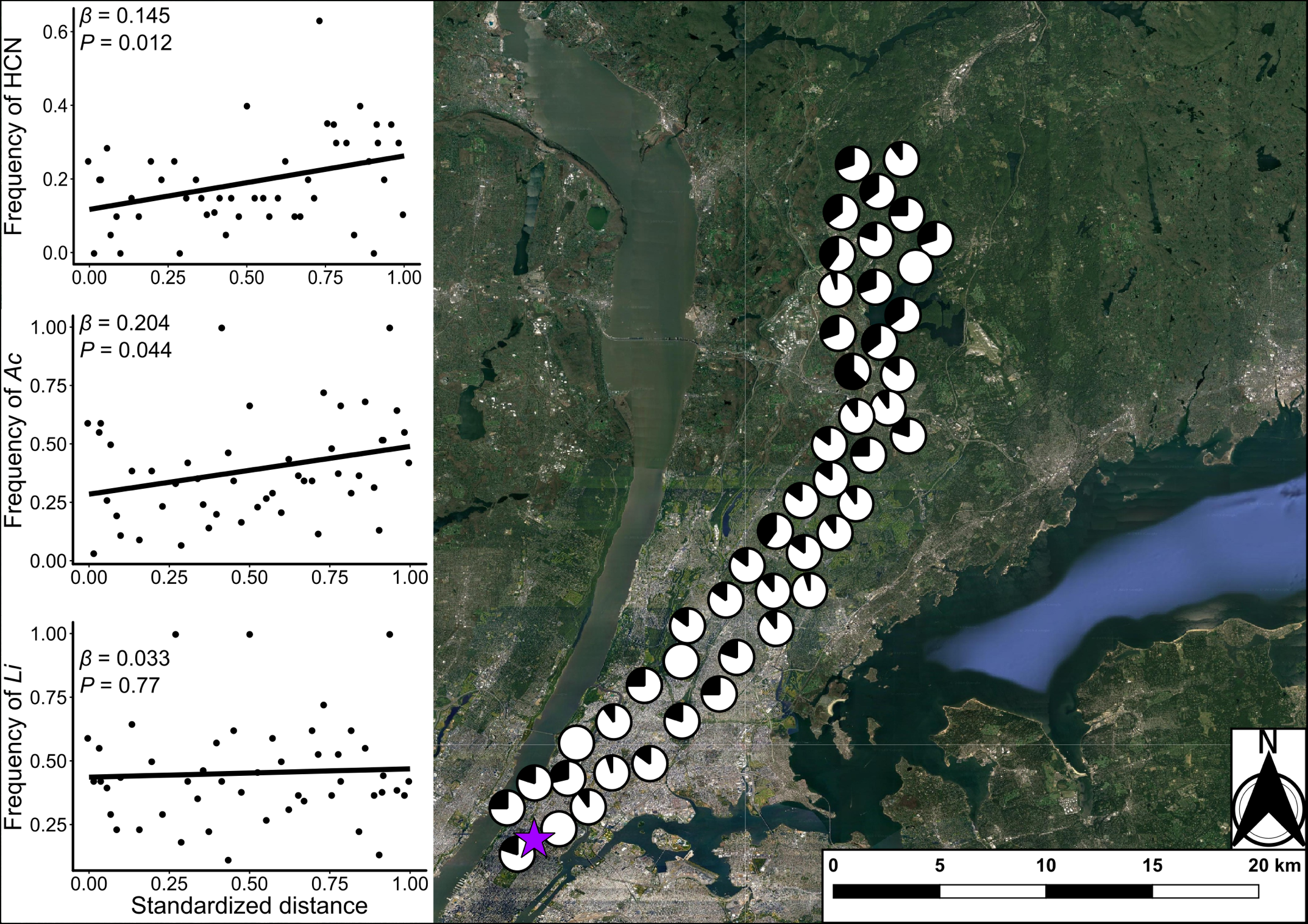


**Figure S17**: Map of the urban-rural transect for the city of New York. Populations along the transect are represented with pie charts showing the proportion of cyanogenic plants (black) in the population. Pie charts have been jittered from their actual location to improve visualization. The purple star represents the location of the city center (Lat: 40.7921, Long: −73.958). Inset shows the best fit regressions for the change in the frequency of HCN, *Ac*, and *Li* along an urbanization gradient, using standardized distance to the city center as a predictor. For each cline, slopes (*β*) and *P*-values for first-order (linear) terms are provided.


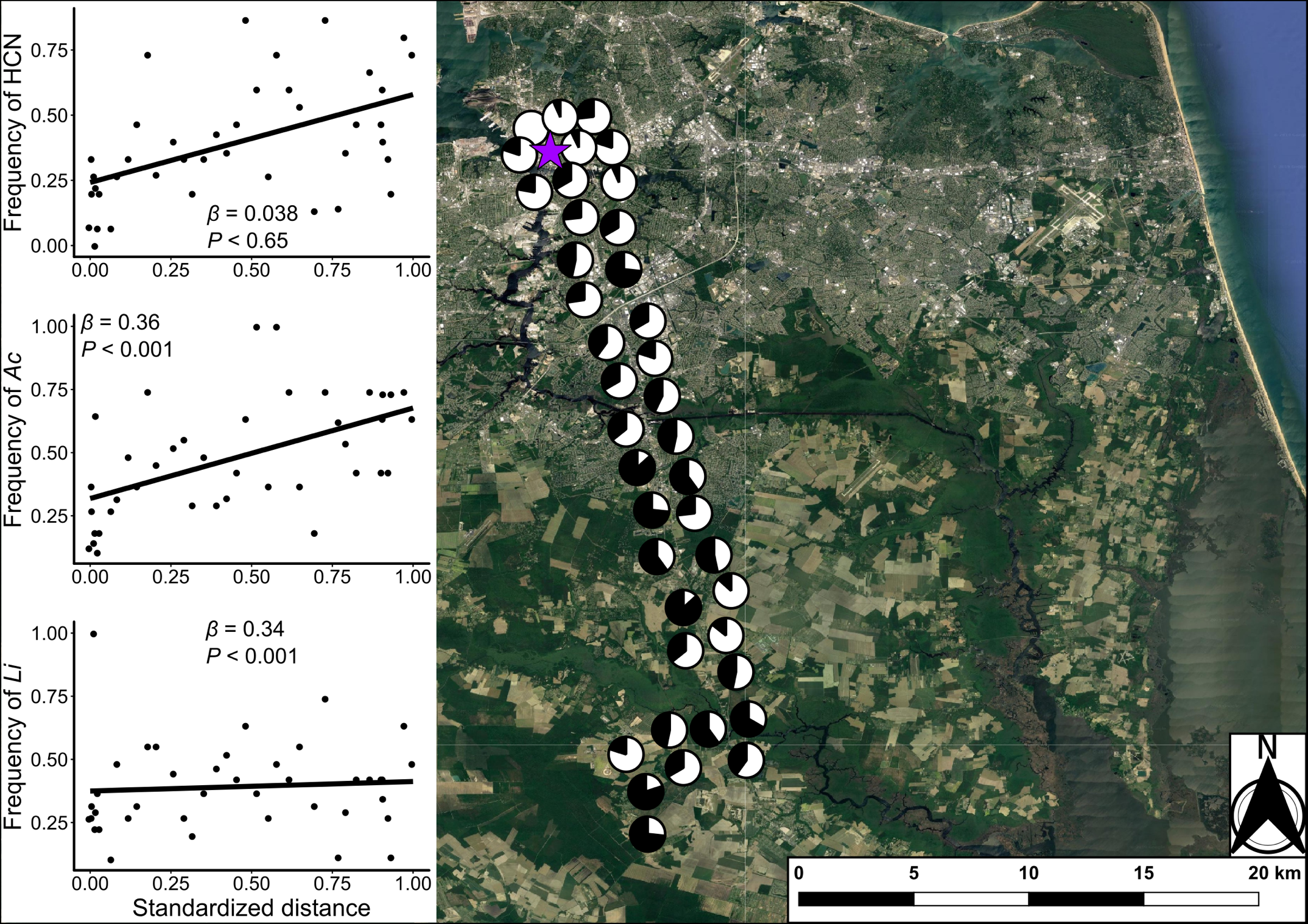


**Figure S18**: Map of the urban-rural transect for the city of Norfolk. Populations along the transect are represented with pie charts showing the proportion of cyanogenic plants (black) in the population. Pie charts have been jittered from their actual location to improve visualization. The purple star represents the location of the city center (Lat: 36.850769, Long: −76.285873). Inset shows the best fit regressions for the change in the frequency of HCN, *Ac*, and *Li* along an urbanization gradient, using standardized distance to the city center as a predictor. For each cline, slopes (*β*) and *P*-values for first-order (linear) terms are provided.


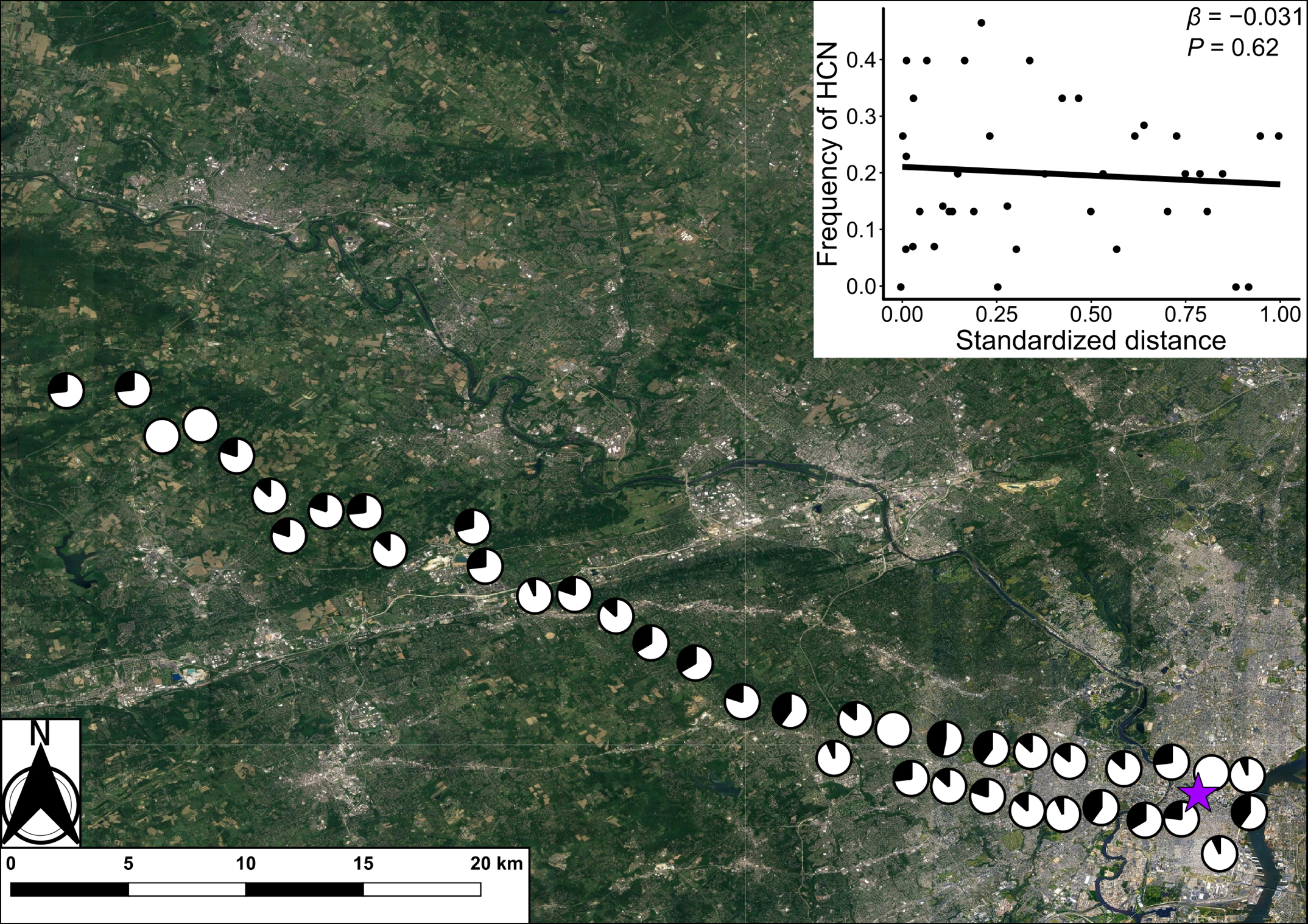


**Figure S19**: Map of the urban-rural transect for the city of Philadelphia. Populations along the transect are represented with pie charts showing the proportion of cyanogenic plants (black) in the population. Pie charts have been jittered from their actual location to improve visualization. The purple star represents the location of the city center (Lat 39.952583, Long: −75.165222). Inset shows the best fit regressions for the change in the frequency of HCN along an urbanization gradient, using standardized distance to the city center as a predictor. The slope (*β*) and *P*-value for first-order (linear) regression is provided.


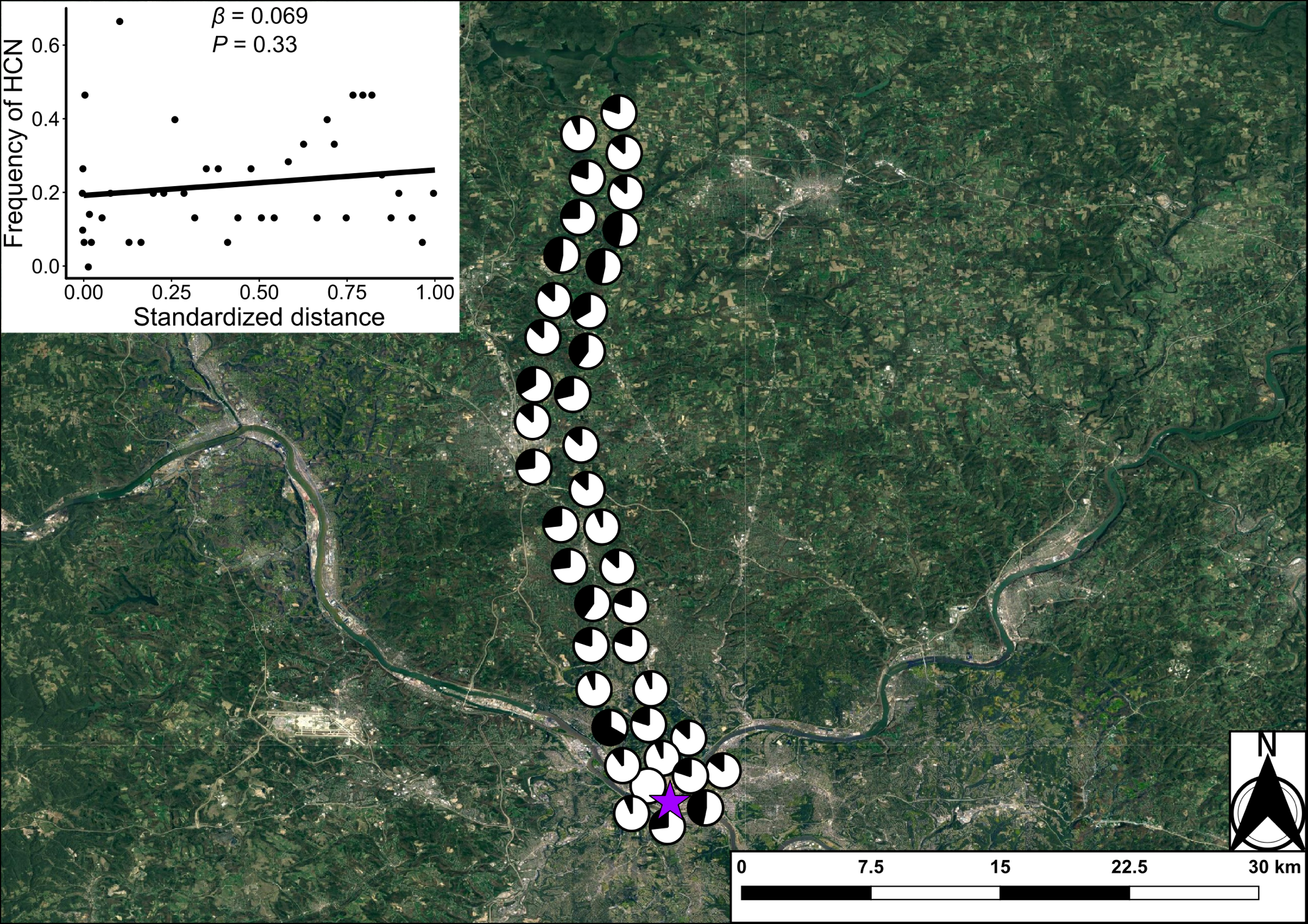


**Figure S20**: Map of the urban-rural transect for the city of Pittsburgh. Populations along the transect are represented with pie charts showing the proportion of cyanogenic plants (black) in the population. Pie charts have been jittered from their actual location to improve visualization. The purple star represents the location of the city center (Lat 40.440624, Long: −79.995888). Inset shows the best fit regressions for the change in the frequency of HCN along an urbanization gradient, using standardized distance to the city center as a predictor. The slope (*β*) and *P*-value for first-order (linear) regression is provided.


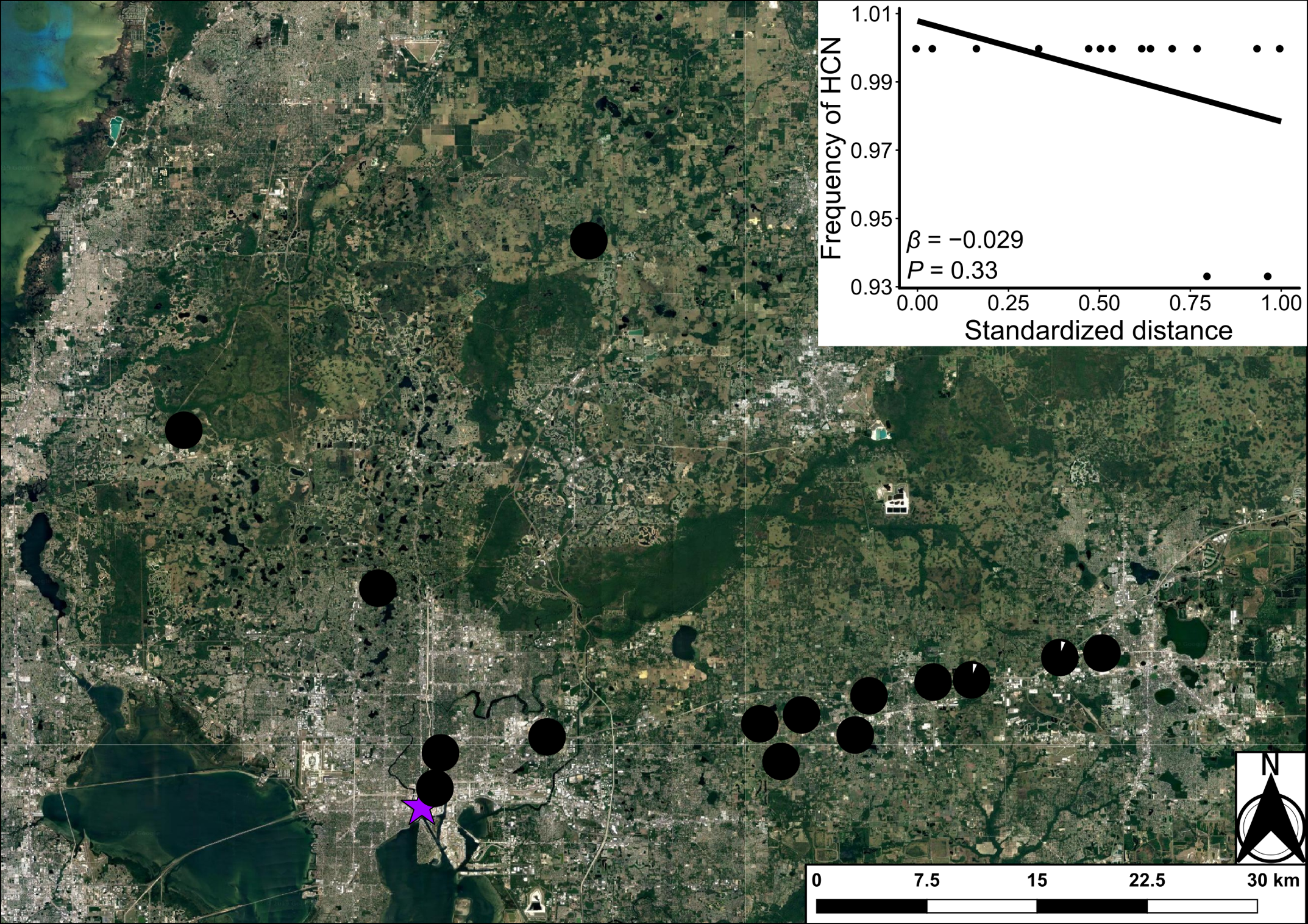


**Figure S21**: Map of the urban-rural transect for the city of Tampa. Populations along the transect are represented with pie charts showing the proportion of cyanogenic plants (black) in the population. Pie charts have been jittered from their actual location to improve visualization. The purple star represents the location of the city center (Lat 27.94742, Long: −82.458778). Inset shows the best fit regressions for the change in the frequency of HCN along an urbanization gradient, using standardized distance to the city center as a predictor. The slope (*β*) and *P*-value for first-order (linear) regression is provided. Note that Tampa was not included in our analysis predicting the strength of clines due to being functionally fixed for cyanogenesis.


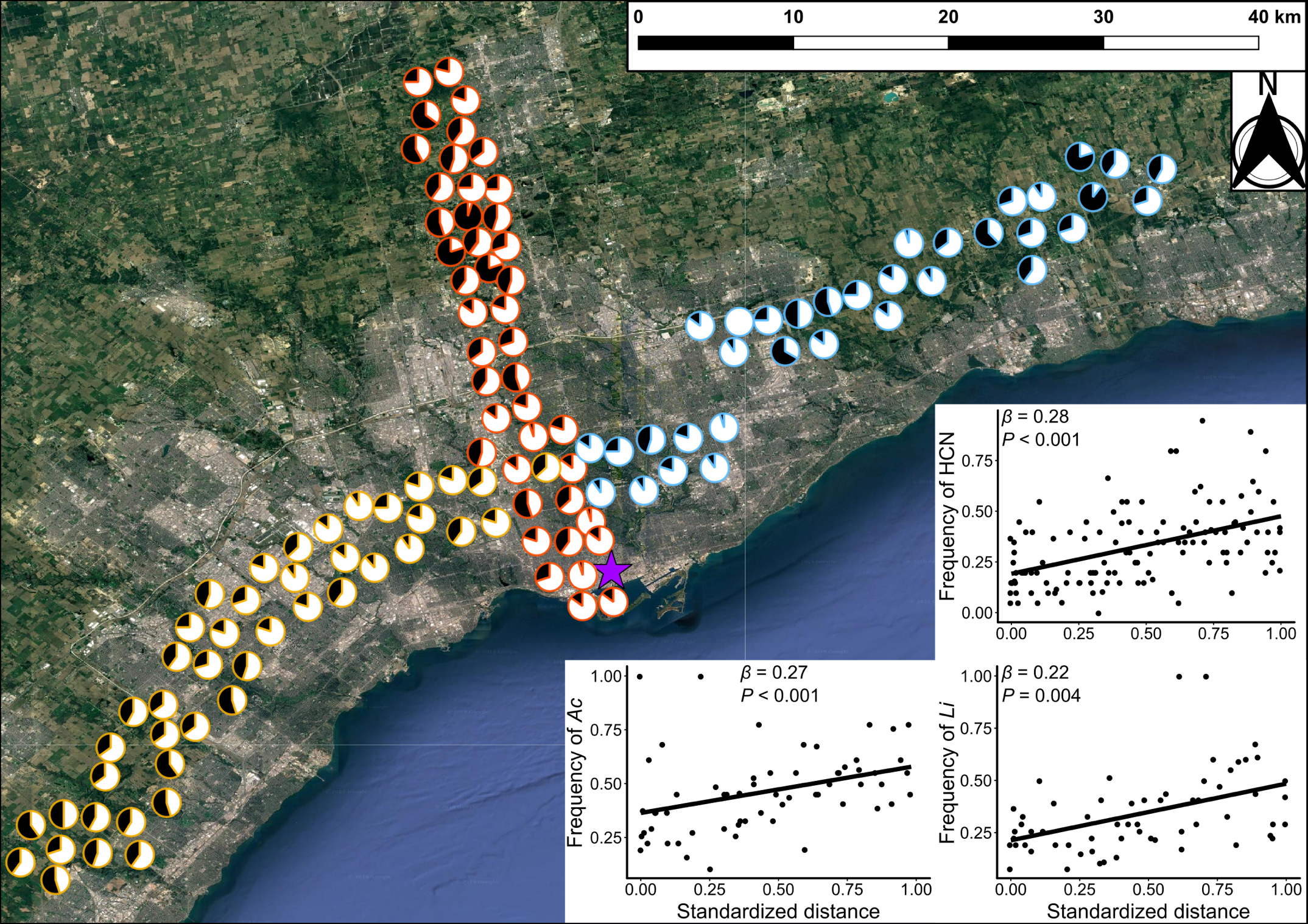


**Figure S22**: Map of the urban-rural transect for the city of Toronto. Populations along the transect are represented with pie charts showing the proportion of cyanogenic plants (black) in the population. Pie charts have been jittered from their actual location to improve visualization. Coloured outlines around pie charts represent the western (yellow), northern (orange), and eastern (blue) transects sampled by Thompson *et al.* (2016). The purple star represents the location of the city center (Lat: 43.6561, Long: −79.3803). Inset shows the best fit regressions for the change in the frequency of HCN, *Ac*, and *Li* along an urbanization gradient, using standardized distance to the city center as a predictor. For each cline, slopes (*β*) and *P*-values for first-order (linear) terms are provided. These regression were run population-means pooled across all transects since each one showed significant clines when analyzed independently.


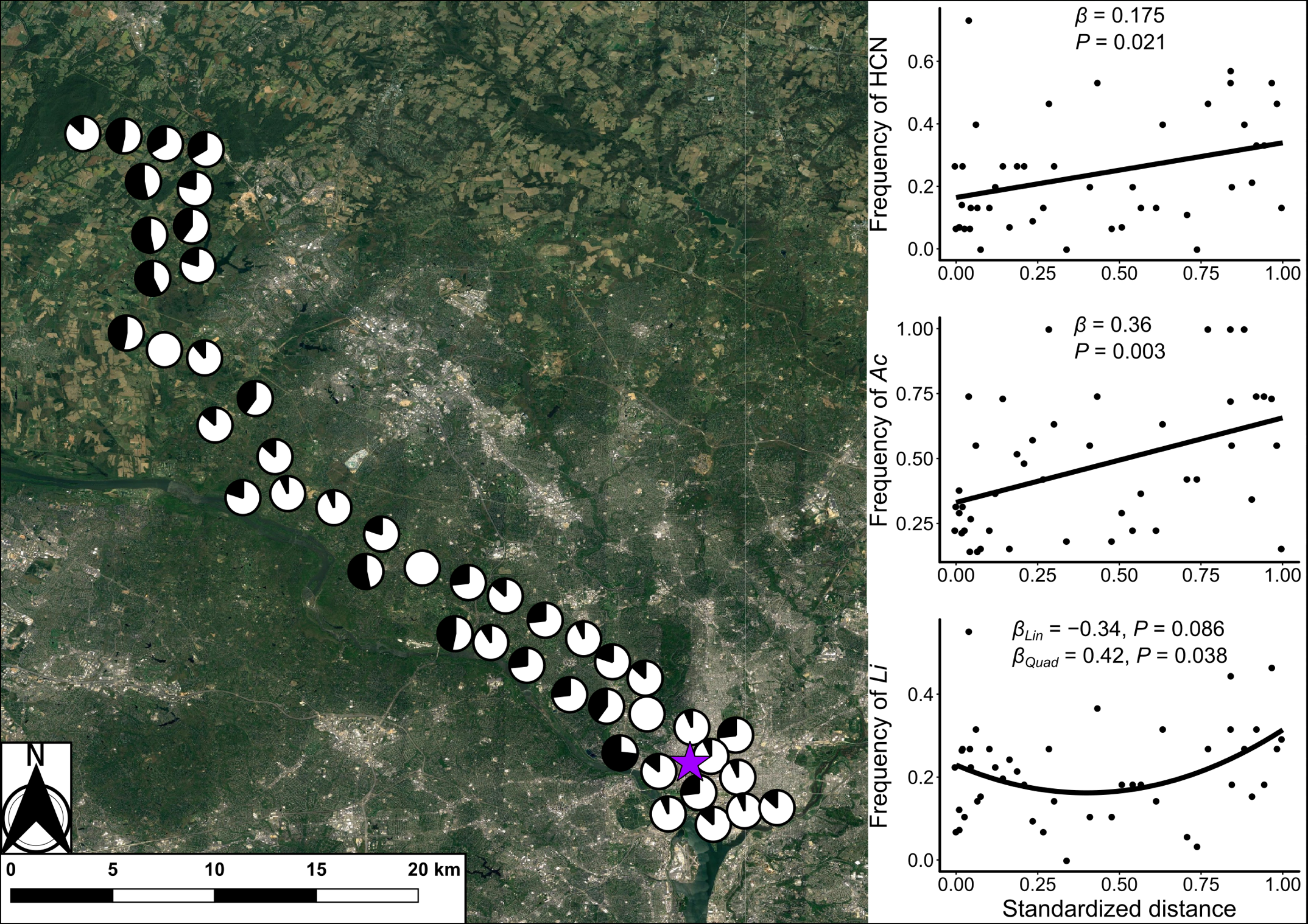


**Figure S23**: Map of the urban-rural transect for the city of Washington D. C. Populations along the transect are represented with pie charts showing the proportion of cyanogenic plants (black) in the population. Pie charts have been jittered from their actual location to improve visualization. The purple star represents the location of the city center (Lat: 38.907192, Long: −77.036873). Inset shows the best fit regressions for the change in the frequency of HCN, *Ac*, and *Li* along an urbanization gradient, using standardized distance to the city center as a predictor. For each cline, slopes (*β*) and *P*-values for first-order (linear) and second-order (quadratic, where applicable) terms are provided.
